# The resonant brain: How attentive conscious seeing regulates action sequences that interact with attentive cognitive learning, recognition, and prediction

**DOI:** 10.3758/s13414-019-01789-2

**Published:** 2019-06-19

**Authors:** Stephen Grossberg

**Affiliations:** Center for Adaptive Systems, Room 213, Graduate Program in Cognitive and Neural Systems, Departments of Mathematics & Statistics, Psychological & Brain Sciences, and Biomedical Engineering, Boston University, 677 Beacon Street, Boston, MA 02215 USA

**Keywords:** Spatial attention, Object attention, Saccadic eye movement, Arm movement, Movement sequences, Complementary computing, Hierarchical resolution of uncertainty, Adaptive resonance, Consciousness, Surface–shroud resonance, Feature–category resonance, Invariant object category learning, Neon color spreading, Boundary completion, Surface filling-in, Figure–ground separation, Cognitive working memory, Cognitive plan, V2, V3A, V4, LIP, IPS, PPC, PFC

## Abstract

This article describes mechanistic links that exist in advanced brains between processes that regulate conscious attention, seeing, and knowing, and those that regulate looking and reaching. These mechanistic links arise from basic properties of brain design principles such as complementary computing, hierarchical resolution of uncertainty, and adaptive resonance. These principles require conscious states to mark perceptual and cognitive representations that are complete, context sensitive, and stable enough to control effective actions. Surface–shroud resonances support conscious seeing and action, whereas feature–category resonances support learning, recognition, and prediction of invariant object categories. Feedback interactions between cortical areas such as peristriate visual cortical areas V2, V3A, and V4, and the lateral intraparietal area (LIP) and inferior parietal sulcus (IPS) of the posterior parietal cortex (PPC) control sequences of saccadic eye movements that foveate salient features of attended objects and thereby drive invariant object category learning. Learned categories can, in turn, prime the objects and features that are attended and searched. These interactions coordinate processes of spatial and object attention, figure–ground separation, predictive remapping, invariant object category learning, and visual search. They create a foundation for learning to control motor-equivalent arm movement sequences, and for storing these sequences in cognitive working memories that can trigger the learning of cognitive plans with which to read out skilled movement sequences. Cognitive–emotional interactions that are regulated by reinforcement learning can then help to select the plans that control actions most likely to acquire valued goal objects in different situations. Many interdisciplinary psychological and neurobiological data about conscious and unconscious behaviors in normal individuals and clinical patients have been explained in terms of these concepts and mechanisms.

## 1. Introduction: How conscious resonant dynamics link perception and cognition to action

This article summarizes a radical departure from the classical view that sensory inputs are transformed via feedforward processes from perception to cognition to action, with little regard for processes of visual attention, memory, learning, decision-making, and interpersonal interaction. Instead, the article summarizes how feedback occurs ubiquitously in our brains to regulate processes of Consciousness, Learning, Expectation, Attention, Resonance, and Synchrony, the so-called CLEARS processes. The CLEARS processes are realized by building upon basic brain designs such as complementary computing, hierarchical resolution of uncertainty, and adaptive resonance that will be described below.

The brain processes that carry out complementary computing and hierarchical resolution of uncertainty clarify not only *how* and *where* conscious states of mind occur in advanced brains but also *why* evolution may have been led to discover conscious states of mind. In brief, conscious states are needed to control the choice of task-relevant actions. This article thus argues that a full understanding of links between cognition and action cannot be achieved without first understanding the fundamental mechanistic link that exists between conscious perceptual and cognitive representations and the choice of effective actions. The article will accordingly describe how a particular hierarchical resolution of uncertainty that occurs in the visual system enables conscious states to be activated that focus spatial attention upon object surfaces. The surface–shroud resonance that sustains spatial attention on an object surface also controls sequences of saccadic eye movements that foveate the object’s salient features. The scanned salient features, in turn, enable learning an invariant object category with which to recognize and predict the object. The foveated positions can also activate reaching movements with which to manipulate the object. Sequences of looking and reaching movements can be stored in working memory, thereby enabling learning of cognitive and motor plans whereby skilled sequential movements can be carried out. Cognitive–emotional interactions help to select the plans that are appropriate in different environments.

These goals cannot be achieved without first understanding how the CLEARS processes contribute to these goals. First and foremost, the CLEARS processes help to solve the *stability–plasticity dilemma*, whereby advanced brains can learn quickly without catastrophically forgetting already learned, but still useful, knowledge at unpredictable times. By solving the stability–plasticity dilemma, humans can rapidly learn enormous amounts of new information, on their own, throughout life, and can integrate all this information into unified conscious experiences that cohere into a sense of self.

Currently popular machine learning algorithms, such as back propagation and deep learning, do experience catastrophic forgetting, in addition to being unable to learn quickly or autonomously in response to a changing world in real time.

Adaptive resonance theory, or ART, solves the stability–plasticity dilemma by showing how the CLEARS processes work together to enable our brains to autonomously learn to attend, recognize, and predict objects and events in a changing world. ART was led to predict that “all conscious states are resonant states” as part of its specification of mechanistic links between the CLEARS processes. These mechanistic links explain data ranging from individual spikes and their synchronization to the dynamics of conscious and unconscious perceptual, cognitive, and cognitive–emotional experiences. ART currently provides unified explanations of much more interdisciplinary data in these areas than other available theories, and all the main ART hypotheses have been supported by subsequent experiments. See Grossberg (2013, 2017b, 2018) for recent expositions.

### Feature–category resonances solve the stability–plasticity dilemma

**Fig. 1:**
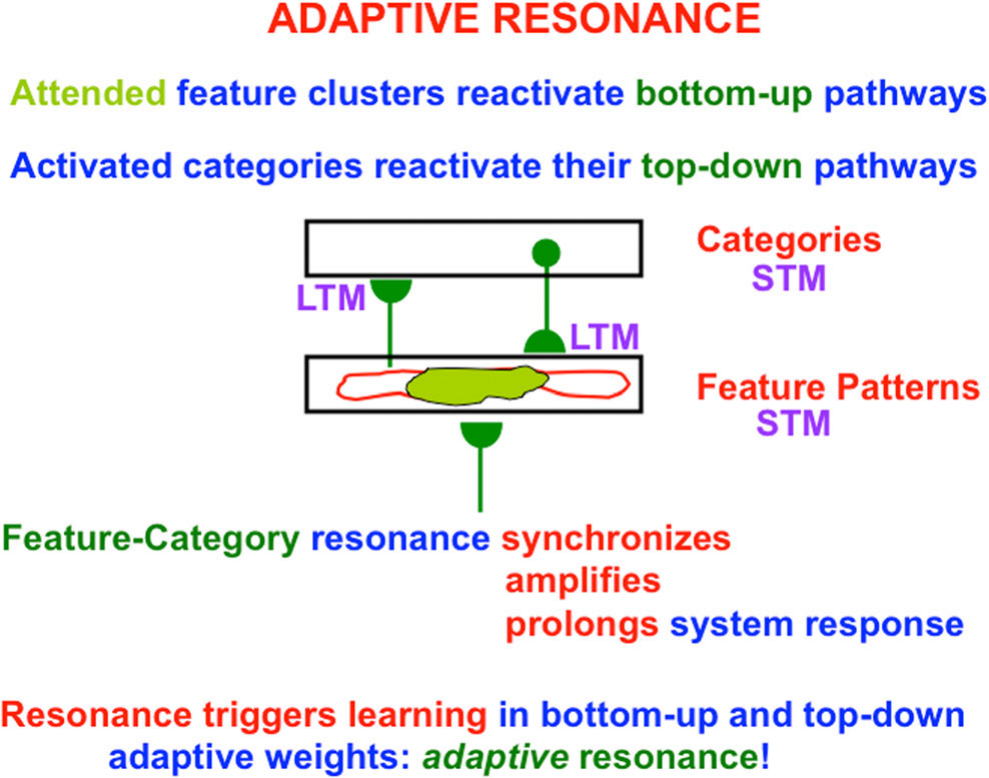
During an adaptive resonance, attended feature patterns interact with recognition categories, both stored in short-term memory (STM), via positive feedback pathways that can synchronize, amplify, and prolong the resonating cell activities. Such a resonance can trigger learning in the adaptive weights, or long-term memory (LTM) traces, within both the bottom-up adaptive filter pathways and the top-down learned expectation pathways. In the present example, the resonance is a feature–category resonance (see Table 1a). (Color figure online)

The CLEARS processes work together as follows to generate *feature–category resonances* (see Fig. 1) in the following way: A bottom-up input pattern activates a distributed pattern of feature-selective cells which, in turn, send bottom-up signals to a category coding level. These bottom-up signals are multiplied by adaptive weights, or long-term memory (LTM) traces, that can be tuned by learning. An activated category then reads out a top-down *expectation*. These top-down signals are also multiplied by LTM traces. These expectations help to focus *attention* upon salient combinations of cues, called critical feature patterns, that are expected in a given environment. If a good enough match occurs between the top-down expectation and a currently active bottom-up feature pattern, then a resonance begins to form between them via the active bottom-up and top-down excitatory pathways.

A *resonance* is a dynamical state during which neuronal firings across a brain network are amplified and *synchronized* when they interact via reciprocal excitatory feedback signals during a matching process that occurs between bottom-up and top-down pathways. Such a resonance can trigger fast *learning* that incorporates the attended critical feature pattern into the LTM traces within the bottom-up adaptive filters that activate recognition categories, and the top-down expectations that are read out by them—hence the name *adaptive* resonance—while suppressing outliers that could have caused catastrophic forgetting, and thereby solving the stability–plasticity dilemma.

### Object attention obeys the ART Matching Rule

The object attentional feedback that enables ART matching to occur obeys the ART Matching Rule, which was predicted to be realized by top-down, modulatory on-center, off-surround networks. Such networks can prime expected feature patterns with their top-down modulatory on-centers, while also inhibiting unexpected features via their off-surrounds. When these top-down selective attention circuits are embodied within the larger neural architectures that the current article describes, they provide a rigorous mechanistic interpretation of concepts like “action-centered attention” (Tipper, Lortie, & Baylis, 1992) and “affordance competition” (Cisek, 2007). Indeed, the affordance competition hypothesis uses the kind of recurrent on-center off-surround networks, also called recurrent competitive fields, from which ART Matching Rule circuits are constructed (Bullock, Cisek, & Grossberg, 1998; Cisek, Grossberg, & Bullock, 1998; Grossberg, 1973).

It has been discovered over the years that adaptive resonances generate parametric properties of individual conscious experiences of seeing, hearing, feeling, and knowing. ART has by now reached sufficient maturity to begin classifying the brain resonances that support conscious awareness during such experiences. Psychological and neurobiological data about conscious and unconscious experiences in both normal individuals and clinical patients have been clarified by this classification (e.g., Franklin & Grossberg, 2017; Grossberg, 2017a, 2017b, 2018; Grossberg & Kishnan, 2018; Grossberg, Palma, & Versace, 2015; Grossberg & Versace, 2008). This analysis also explains why not all resonances become conscious, and why not all brain dynamics are resonant, as discussed in Sections 3 and 4 below.

Sections 2 and 4 will summarize the fact that many advanced neocortical systems are organized into pairs of parallel processing streams that obey computationally complementary laws. The streams interact together using multiple processing stages to overcome the uncertainties that each stream, acting alone, would face. As noted above, such a hierarchical resolution of uncertainty clarifies why the evolutionary process was driven to discover conscious states upon which reliable actions could be based. Sections 5 and 6 will review complementary properties of perceptual/cognitive processes in the ventral “what” cortical stream and spatial/action processes in the dorsal “where” cortical stream. Sections 7 and 8 will describe the hierarchical resolutions of uncertainty that occur in the visual system in order to compute the boundary and surface representations that can be used for seeing, recognition, and action. Sections 9 and 10 summarize the surface–shroud resonances and feature–category resonances that build upon these processes. Sections 11 and 12 explain how invariant object categories are learned during free scanning of a scene, including how sequences of eye movements are generated to foveate salient features of different object views as invariant learning takes place. Section 13 summarizes how this foundation supports motor-equivalent sequences of arm movements to these salient features, and how these sequences may be stored within the prefrontal cortex in cognitive working memories that trigger the learning of cognitive plans. When modulated by cognitive–emotional interactions that are sculpted during reinforcement learning and incentive motivational learning, these cognitive plans may be used to choose the movements that will most probably acquire valued goals in different environments.

## 2. Why was evolution led to discover conscious states? Hierarchical resolution of uncertainty

ART goes beyond explanations of how, where, and when conscious states may be generated within our brains. It also proposes *why* evolution may have been driven to discover conscious states in the first place. This explanation follows naturally from the design principles that our brains use to autonomously adapt in real time to a changing world that may be filled with unexpected events. One of these design principles is called the *hierarchical resolution of uncertainty*. Hierarchical resolution of uncertainty means that it often takes multiple processing stages for our brains to generate a sufficiently complete, context-sensitive, and stable perceptual representation upon which to base a successful action.

**Fig. 2:**
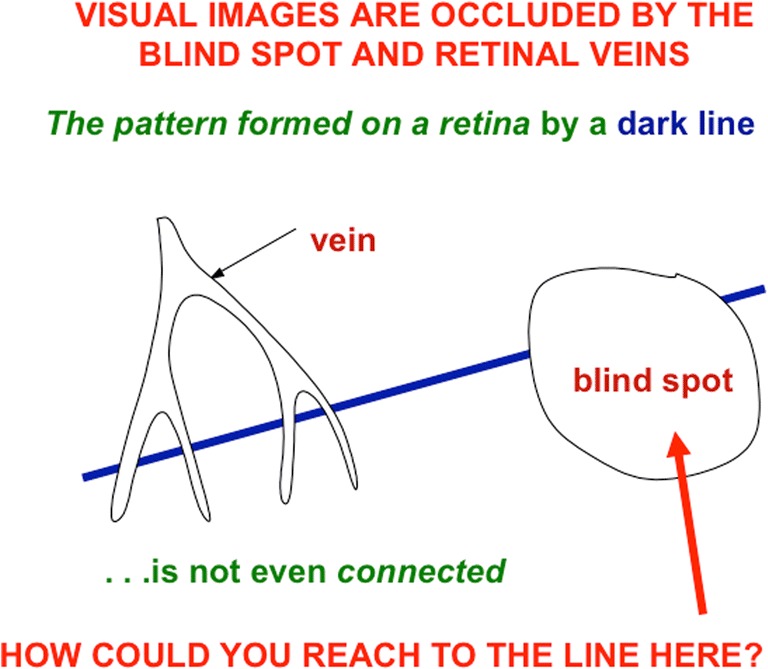
This image emphasizes that, even the retinal image of a simple object like a line can be occluded in multiple places by retinal veins and the blind spot, thereby creating multiple positions along the line that do not provide reliable inputs to the brain for directing actions to those positions. (Color figure online)

For example, during vision, light strikes the photosensitive retina. However, there is a large blind hole in the retina that is called the retinal blind spot where no light is registered. This hole is there because it is where all the photoreceptors send their axons to be bundled into the optic nerve on their way to the brain. In addition, the light is occluded by retinal veins that nourish retinal cells, and passes through all the other retinal layers before it ever hits the light-sensitive photoreceptor layer. Thus, even a rather complete scene in the world under good lighting conditions is registered by retinal photoreceptors as an occluded and noisy image in each eye. Figure 2 illustrates this problem with the simple example of a line that is registered on the retina through positions that intersect the blind spot and retinal veins. One can readily see from this example that it could be highly problematic to use such incomplete and noisy data to choose a reaching movement to a position on the line where, say, it is occluded by the blind spot.

Multiple processing stages are needed to complete 3-D boundary and surface representations with which to more informatively represent the scene (e.g., Grossberg, 2017b). Doing so requires that three hierarchical resolutions of uncertainty occur, which will be described below.

ART predicts that the processing stage where such a sufficiently complete, context-sensitive, and stable surface representation is completed “lights up” into a conscious state due to a resonance with a subsequent processing stage that marks this surface representation as being a good enough one upon which to base a successful action of looking or reaching. Such a resonance is called a *surface–shroud resonance* because the completed representation is a surface representation, and the form-fitting spatial attentional representation that resonates with it is called an *attentional shroud* (Fazl, Grossberg, & Mingolla, 2009; Tyler & Kontsevich, 1995). Surface–shroud resonances are predicted to be triggered by interactions between prestriate visual cortical area V4 and the posterior parietal cortex (PPC), before propagating both top-down to lower cortical areas such as V2 and V1, and bottom-up to higher cortical areas such as prefrontal cortex (PFC). Had earlier processing stages been used to trigger these actions, the wrong actions could have been generated, with potentially disastrous consequences for survival. This conscious state hereby provides an “extra degree of freedom” that enables our brains to avoid prematurely generating responses using inadequate perceptual representations.

In this way, ART clarifies that there is an intimate link between conscious states of seeing, hearing, feeling, and knowing, and the choice and execution of context-appropriate actions. ART proposes how resonances for conscious seeing help to ensure effective looking and reaching, resonances for conscious hearing help to ensure effective communications including speaking, and resonances for conscious feeling help to ensure effective goal-directed actions. ART also proposes how, when we consciously see a familiar valued object, we can also know some things about it, and have appropriate feelings about it.

The PPC can be both a source of top-down spatial attention with which to resonate with visual surface representations during a surface–shroud resonance, and of bottom-up motor commands to move the eyes and arms to attended positions in space, leading to the distinction between *attention* and *intention* in descriptions of parietal function (e.g., Andersen, Essick, & Siegel, 1985; Gnadt & Andersen, 1988; Snyder, Batista, & Andersen, 1997, 1998, 2000).

ART has classified six different types of neural representations of conscious qualia (see Table 1a; Grossberg, 2017b). This article will summarize how visual perceptual and cognitive consciousness are linked to actions by normal individuals and those with visual agnosia.

**Table 1:**
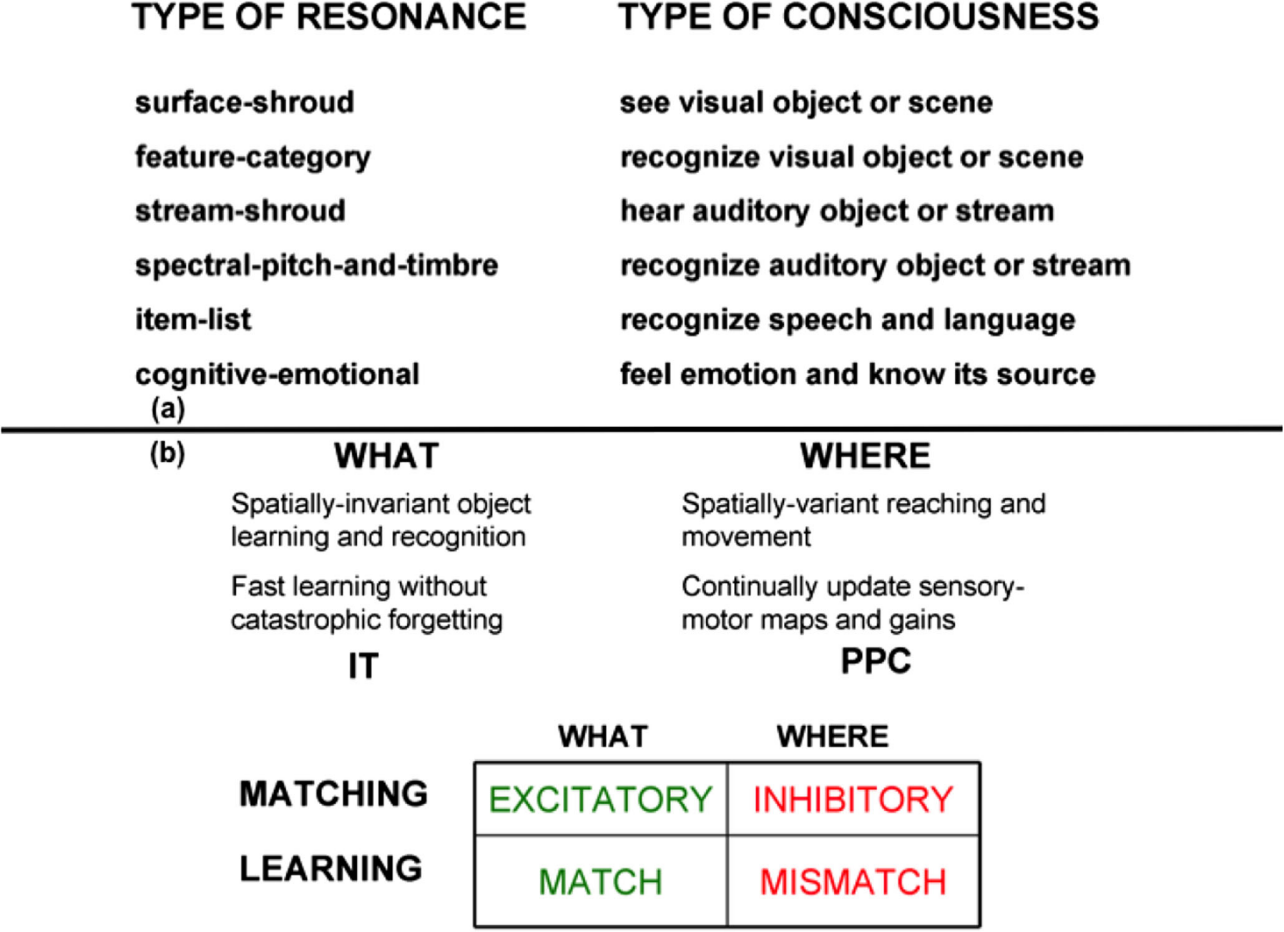
**a** Types of resonances and the conscious experiences that they embody. **b** Complementary “what” and “where” cortical stream properties. Cortical “what” stream perceptual and cognitive representations can solve the stability–plasticity dilemma, using brain regions like inferotemporal (IT) cortex, where recognition categories are learned. These processes carry out excitatory matching and match-based learning. Cortical “where” stream spatial and motor processes often carry out inhibitory matching and mismatch-based learning that do not solve the stability–plasticity dilemma, but rather adapt to changing bodily parameters, using brain regions like posterior parietal cortex (PPC). Whereas the recognition categories in the cortical “what” stream become increasingly invariant at higher cortical levels with respect to object views, positions, and sizes, the cortical “where” stream elaborates spatial representations of object positions and mechanisms whereby to act upon them. Together, the two streams can learn to recognize and become conscious of valued objects and scenes, while directing appropriate actions towards them

## 3. All conscious states are resonant states, but not conversely

Although ART predicts that “all conscious states are resonant states,” it does not predict that “all resonant states are conscious states.” Resonant states that are not accessible to consciousness, but that nonetheless dynamically stabilize learned memories, include parietal-prefrontal resonances that trigger the selective opening of basal ganglia gates to enable the readout of contextually appropriate thoughts and actions (Brown, Bullock, & Grossberg, 2004; Buschman & Miller, 2007; Grossberg, 2016b) and entorhinal-hippocampal resonances that dynamically stabilize the learning of entorhinal grid cells and hippocampal place cells during spatial navigation (Grossberg & Pilly, 2014; Kentros, Agniotri, Streater, Hawkins, & Kandel, 2004; Morris & Frey, 1997; Pilly & Grossberg, 2012). These resonances do not include feature detectors that are activated by external senses—such as those that support vision or audition—or internal senses—such as those that support emotion. Hence, they cannot become conscious.

## 4. Complementary computing and hierarchical resolution of uncertainty

Another reason why not all brain dynamics may lead to conscious states is that not all brain dynamics can become resonant, notably, spatial and motor processes, a property that is relevant for understanding how conscious perception and cognition are linked to action. The fact that not all brain dynamics are resonant is due to *complementary computing* (Grossberg, 2000, 2013, 2017b).

Complementary computing concerns the discovery that pairs of parallel cortical processing streams compute computationally complementary properties in the brain. The existence of processing streams is consistent with the idea that brain processing is specialized, but it does not imply that these streams contain independent modules. For example, Cavanagh (1986) has described independent modules for luminance, motion, binocular disparity, color, and texture that are combined together into more complex visual attributes at higher cortical processing stages. Independent modules should be able to fully compute their particular processes on their own. Much perceptual data argue against such independence. In particular, changes in perceived form or color can cause changes in perceived motion, and conversely. Changes in perceived brightness can cause changes in perceived depth, and conversely. For example, making an object in a picture brighter can make it look closer, relative to other objects in the scene, a property that is often called *proximity-luminance covariance* (Dosher, Sperling, & Wurst, 1986; Schwartz & Sperling, 1983).

Complementary computing explains such strong interactions between perceptual qualities by showing that each cortical processing stream has complementary computational strengths and weaknesses. These streams overcome their complementary deficiencies by interacting with one another using multiple processing stages that realize a hierarchical resolution of uncertainty, leading to perceptual representations that overcome the complementary uncertainties that each stream, on its own, would compute. The result is sufficiently complete, context-sensitive, and stable enough representations upon which successful actions can be based.

## 5. Complementary perceptual/cognitive and spatial/action streams: Tying cognition to action

Table 1b summarizes basic complementary properties of the “what” cortical stream for perception and cognition, and of the “where” cortical stream for spatial representation and action (Mishkin, 1982; Mishkin, Ungerleider, & Macko, 1983). Perceptual/cognitive processes in the “what” stream, which include the inferotemporal cortex, or IT, often use ART-like *excitatory matching* and *match-based learning* to create self-stabilizing categorical representations of objects and events that solve the stability–plasticity dilemma. An example of excitatory matching is that if you are primed to expect to see a yellow ball in a certain place, then you can recognize it more quickly and vigorously than if you were not primed. These excitatory matching and match-based learning processes enable increasing expertise, and an ever-expanding sense of self, to be rapidly and stably learned throughout life.

Table 1b also summarizes that complementary spatial/motor processes in the “where” stream, which include the posterior parietal cortex, or PPC, often use *inhibitory matching* and *mismatch-based learning* to continually update spatial maps and motor controllers that enable our changing bodies to carry out appropriate actions throughout life. This kind of inhibitory processing is often called Vector Associative Map, or VAM, processing (Gaudiano & Grossberg, 1991, 1992). Inhibitory matching subtracts an outflow representation of where our arm is now in space—a *present position vector*—from one that computes the position where we want to move—a *target position vector*—to compute a representation of the direction and distance of a desired movement—a *difference vector* (Bullock & Grossberg, 1988; Everts & Tanji, 1974; Georgopoulos, Kalaska, Caminiti, & Massey, 1982; Georgopoulos, Schwartz, & Kettner, 1986; Kalaska, Caminiti, & Georgopoulos, 1983). When the arm reaches the position where we want it to be, the target and present position vectors both code the same position in space, so the difference vector equals zero.

This kind of inhibitory matching cannot solve the stability–plasticity dilemma for two kinds of reasons. First, an inhibitory match cannot support an excitatory resonance, and thus cannot dynamically stabilize its learned representations using a resonant mechanism. Second, VAM mismatch learning calibrates a target position vector to equal the present position vector that is active when they both represent the same position in space. Thus, whenever bodily relationships change throughout life due to development, growth, exercise, and aging, the new present positions that are generated as a result will recode the corresponding target positions. Thus, spatial and motor learning experience continual overwriting by new experiences so that our brains can continue to learn how to accurately move our bodies as they change throughout life. Because they cannot resonate, spatial and motor representations, which are often called *procedural memories* (N. J. Cohen & Squire, 1980; Mishkin, 1982; Scoville & Milner, 1957; Squire & Cohen, 1984), cannot generate conscious internal representations; that is, there are no motor “qualia” that consciously represent the target and present positions of a planned action, even though we can consciously will the action to occur by choosing a target position or learned plan to execute a motor skill, and execute it by activating a volitional GO signal, as Section 13 explains in greater detail.

## 6. Invariant object category learning, Where/How stream, and reaching with visual form agnosia

An important reason for the “what–where” complementarity summarized in Table 1b is that the cortical “what” stream learns object recognition categories that become substantially invariant under changes in an object's view, size, and position at higher cortical processing stages, such as at the anterior inferotemporal cortex (ITa) and beyond (e.g., Booth & Rolls, 1998; Cao, Grossberg, & Markowitz, 2011; Chang, Grossberg, & Cao, 2014; Fazl et al., 2009; Tanaka, 1997, 2000). These invariant object categories enable our brains to recognize valued objects without experiencing the combinatorial explosion that would occur if they needed to store every individual experience, or exemplar, of every familiar object. However, because they are spatially invariant, these categories cannot locate and act upon a desired object in space. Cortical “where” stream spatial and motor representations can locate objects and trigger actions toward them, but cannot recognize them. By interacting together, the “what” and “where” streams can consciously see and recognize valued objects *and* direct appropriate goal-oriented actions toward them. Complementary computing hereby clarifies classical data that the cortical “where” stream is also a cortical “how” stream for the control of action, and is thus often called the “where/how” stream (Goodale & Milner, 1992; Goodale, Milner, Jakobson, & Carey, 1991). In particular, a top-down cognitive prime from the prefrontal cortex of the “what” cortical stream can bias how spatial attention is allocated in the “where” cortical stream and, with it, the actions that are thereby triggered (e.g., Baldauf & Desimone, 2014; Bichot, Heard, & DeGennaro, 2015; Fuster, 1973; Grossberg, 2018).

Studies of how these streams interact have clarified how some actions can occur without conscious knowledge of the objects to which they are directed. This occurs, for example, during visual form agnosia. The famous agnostic patient, D. F., was reported by Milner et al. (1991; see also Binstead, Brownell, Vorontsova, Heath, & Sauciser, 2007; Milner & Goodale, 1995). When D. F. visually inspected an oriented slot, her reports of the orientation of the slot showed little relationship to its actual orientation, whether her reports were made verbally or manually. However, when D. F. was asked to insert her hand, or a hand-held card, into the slot, D. F. did so accurately. In addition, her hand began to rotate in the appropriate direction as soon as it left the start position. In summary, although D. F. did not “know” the orientation of the slot, she could “see” the slot and insert her hand, or post a card into it, with considerable skill. How this can happen will also be explained below.

## 7. Three hierarchical resolutions of uncertainty to complete visual boundaries and surfaces

In order to understand how a surface–shroud resonance can support conscious seeing of visual qualia (see Table 1a) and thus be able to look at and reach attended objects, it is necessary to summarize basic cortical processes about how the brain sees. Perhaps the most basic fact about 3-D vision and figure–ground perception is that its functional units are 3-D *boundaries* and *surfaces*, processes that were first modeled in Grossberg (1984) and have enabled subsequent explanations and predictions of many data, including how looking at 2-D pictures can generate conscious 3-D percepts of occluding and occluded objects (e.g., Cao & Grossberg, 2005; Fang & Grossberg, 2009; Grossberg, 1994, 1997, 2016a; Grossberg & Yazdanbakhsh, 2005; Kelly & Grossberg, 2000), whose properties will be shown in Sections 11, 12, and 13 to be important for control of looking and reaching.

**Fig. 3:**
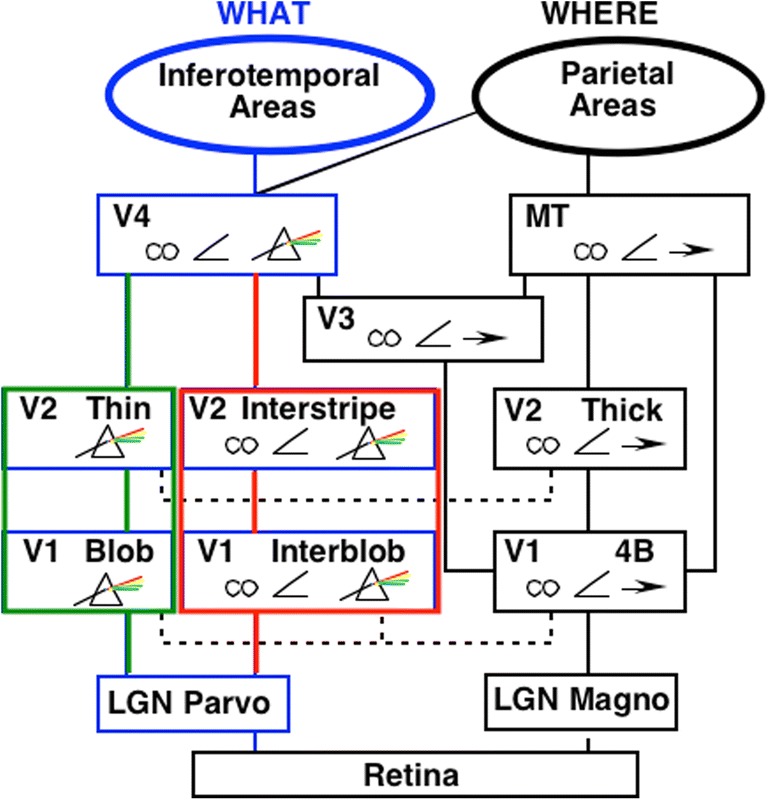
Simplified schematic of the anatomy of three processing streams in the visual cortex. The LGN-blob-(thin stripe)-V4 stream fills-in visual surfaces, whereas the LGN-interblob-interstripe-V4 stream completes visual boundaries. LGN = lateral geniculate nucleus; V1 = striate visual cortex; V2, V3, V4, MT = prestriate cortical areas. The motion stream goes through V1 and MT to the parietal areas. Reproduced with permission from DeYoe and van Essen (1988). (Color figure online)

**Fig. 4:**
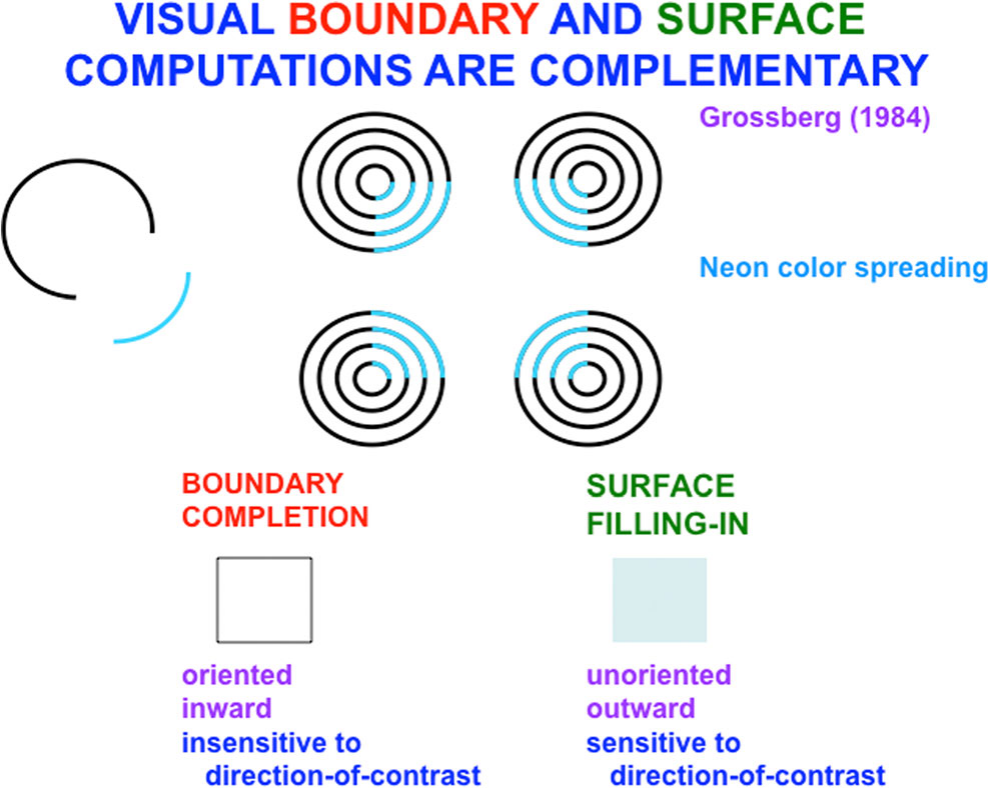
An example of neon color spreading. The image consists of black and blue circular arcs. The percept of the color blue filling a square is a visual illusion that is constructed by the brain. The process whereby boundaries are completed are computationally complementary to the process whereby surfaces fill-in brightness, color, and depth. See the text for details. (Color figure online)

Visual boundaries and surfaces are computed by the interblob and blob cortical processing streams, respectively, that occur within and between cortical areas V1, V2, and V4 (see Fig. 3), and provide examples of both complementary computing and hierarchical resolution of uncertainty. The visual illusion of neon color spreading vividly illustrates the complementary properties of how boundaries are completed and surface brightnesses and colors are filled in (see Fig. 4).

### Neon color spreading, end gaps, and end cuts

Neon color spreading was reported in Varin (1971), who studied a “chromatic spreading” effect that was seen when viewing an image like the one in Fig. 4. Van Tuijl (1975) called related images examples of “neon-like color spreading.” Each black arc and blue arc in Fig. 4 generates boundaries in our brains. At the positions where these boundaries touch, the boundaries caused by black arcs cause small breaks, called *end gaps*, to occur in the boundaries caused by blue arcs *if* the contrast of the black arcs with respect to the white background is larger than the contrast of the blue arcs with respect to the white background.

**Fig. 5:**
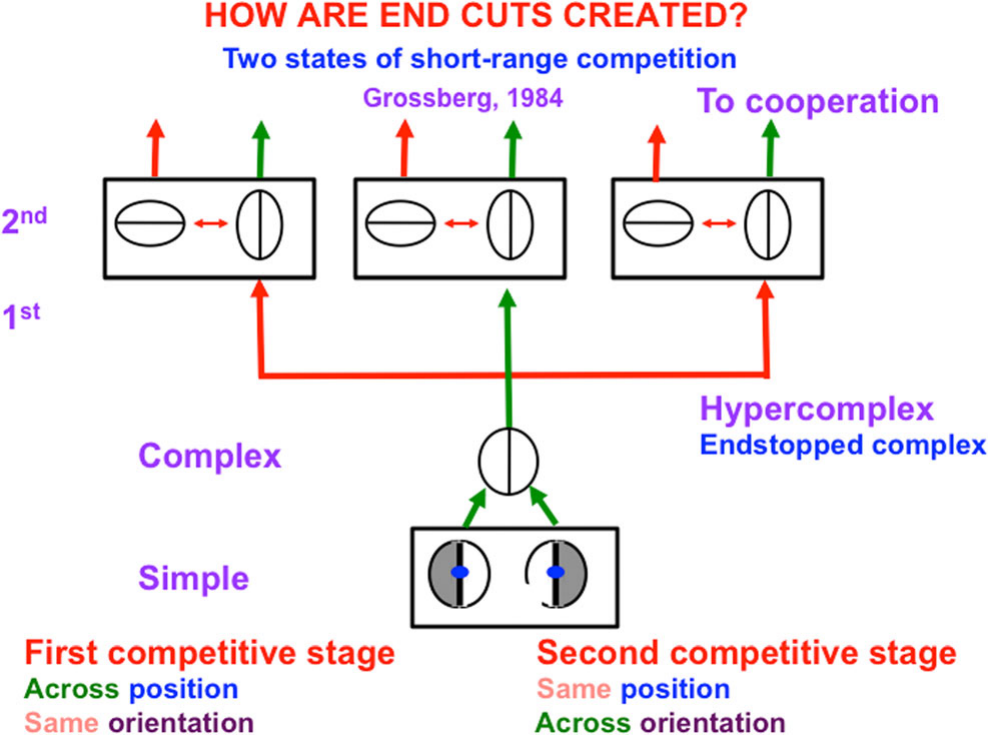
A network of simple, complex, and hypercomplex cells begins the processing of perceptual boundaries. Pairs of like-oriented simple cells with opposite contrast polarity selectivities at each position add their inputs to complex cells. Complex cells input to hypercomplex cells through a short range spatial competition (1st), followed by an orientational competition at each position (2nd). The spatial competition can cause end gaps in boundaries. The orientational competition can cause end cuts in boundaries. The hypercomplex cells in the second competitive stage input to cooperative bipole grouping cells. (Color figure online)

End gaps are created in the following way: The boundary cells that are activated by the image in Fig. 4 are contrast-sensitive and orientationally tuned. These include the simple cells and complex cells in Fig. 5. Both the simple and complex cells that are activated by the black–white image contrasts become more active than the cells that are activated by blue–white contrasts. The active complex cells excite hypercomplex cells at their own positions at the next processing stage, while inhibiting neighboring hypercomplex cells via a short-range *spatial competition* network (see Fig. 5). Due to the contrast sensitivity of hypercomplex cell responses, the stronger black–white boundary signals inhibit nearby blue–white boundary cells more than conversely, thereby weakening the contiguous blue–white boundary—that is, creating an *end gap*.

The boundary cells at the hypercomplex level where end gaps form are tonically active and inhibit other boundary cells that are tuned to different orientations at the same position (see Fig. 5)—that is, by an *orientational competition*. In the absence of external inputs, the tonic activity of these cells is held in check by their mutual competition. When blue–white boundary cells are inhibited, the competitive balance is upset, causing cells that are tuned to other orientations, notably, the perpendicular orientation, to be disinhibited and to thereby create an extra boundary segment that is called an *end cut*.

In summary, end gaps and end cuts are formed as a result of two successive stages of spatial and orientational competition between contrast-sensitive and orientationally tuned hypercomplex cells (Grossberg, 1984; Grossberg & Mingolla, 1985).

### Simple cells cannot detect line ends, but hypercomplex cells can

**Fig. 6:**
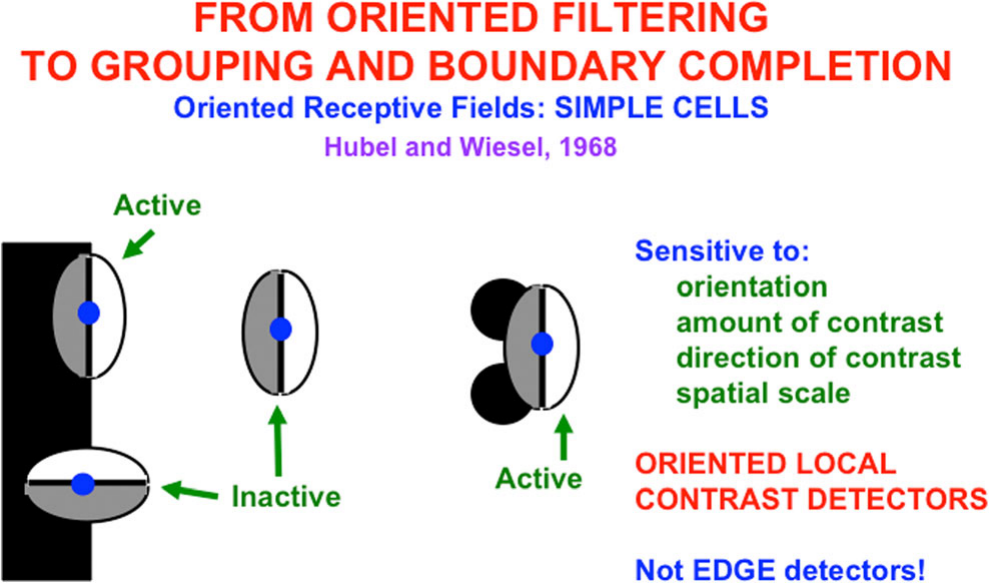
Simple cells are oriented local contrast detectors that are sensitive to the position, orientation, amount and direction of contrast, and size of visual stimuli. They are not just edge detectors, but can rather response to oriented edges, shading, and texture. (Color figure online)

Why does the brain compute end cuts and end gaps at hypercomplex cells? This is an example of a hierarchical resolution of uncertainty that compensates for ambiguous responses of the simple and complex cells that input to hypercomplex cells. Simple cells begins to estimate boundary orientations at each position (Hubel & Wiesel, 1968). They can respond to an oriented distribution of contrasts in response to scenic lines, edges, textures, and shading, not just edges alone (see Fig. 6).

**Fig. 7:**
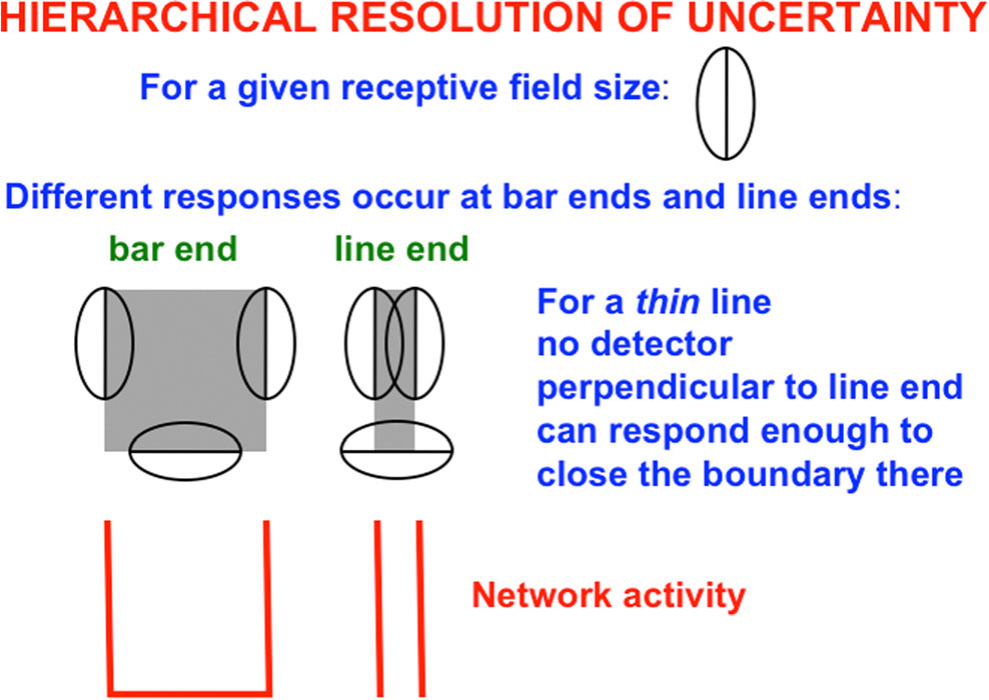
Although simple cells with the proper contrast polarity and orientational selectivity can respond to all sides of a sufficiently thick bar, they cannot respond to the ends of sufficiently thin lines. For each spatial scale of a simple cell, one can construct a range of line widths that has this property. The red lines represent the positions and orientations of the simple cell responses to the bar and line end in the image. (Color figure online)

Simple cells cannot, however, respond at the ends of sufficiently thin lines (see Fig. 7). Without additional boundary processing, gaps would exist in boundaries at line ends. Brightness and color could flow through these gaps via surface filling-in (see Fig. 4). Every scene that contains line ends would thus overflow with spurious filling-in of brightness and color.

**Fig. 8:**
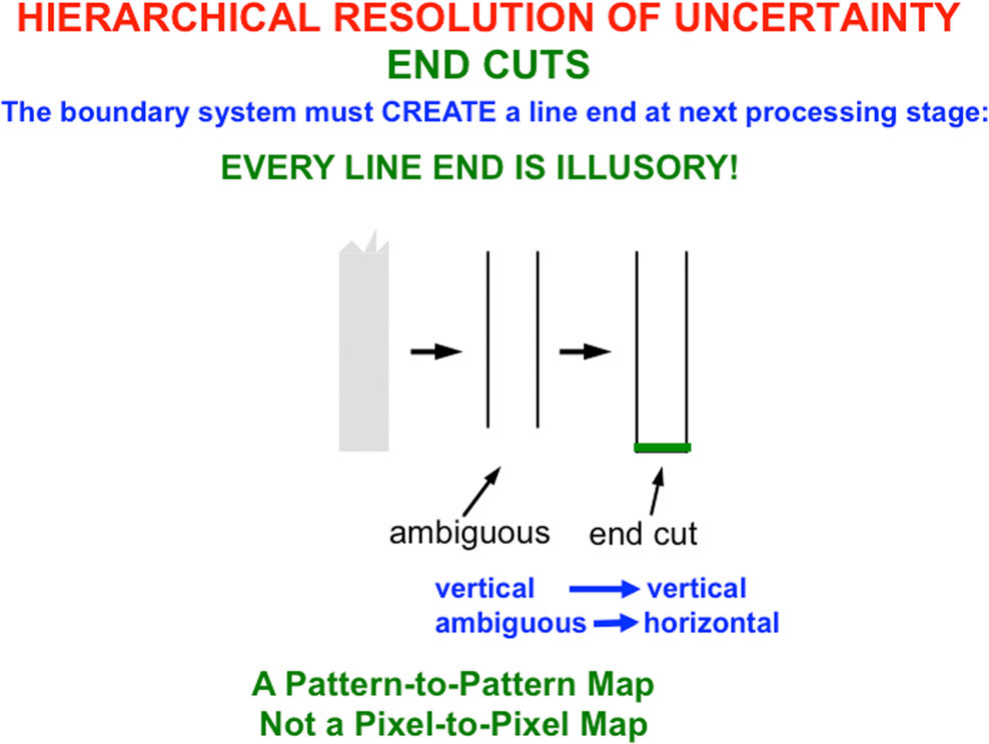
The missing boundary at the bottom of the line end is completed by an end cut. The brain network that does this needs to be sensitive to the *pattern* of activations near the line end, not just to the responses or nonresponses of cells to individual positions, or pixels. Otherwise, the brain would be faced with the impossible task of creating something (an end cut) out of nothing (the nonresponse at the line end). (Color figure online)

**Fig. 9:**
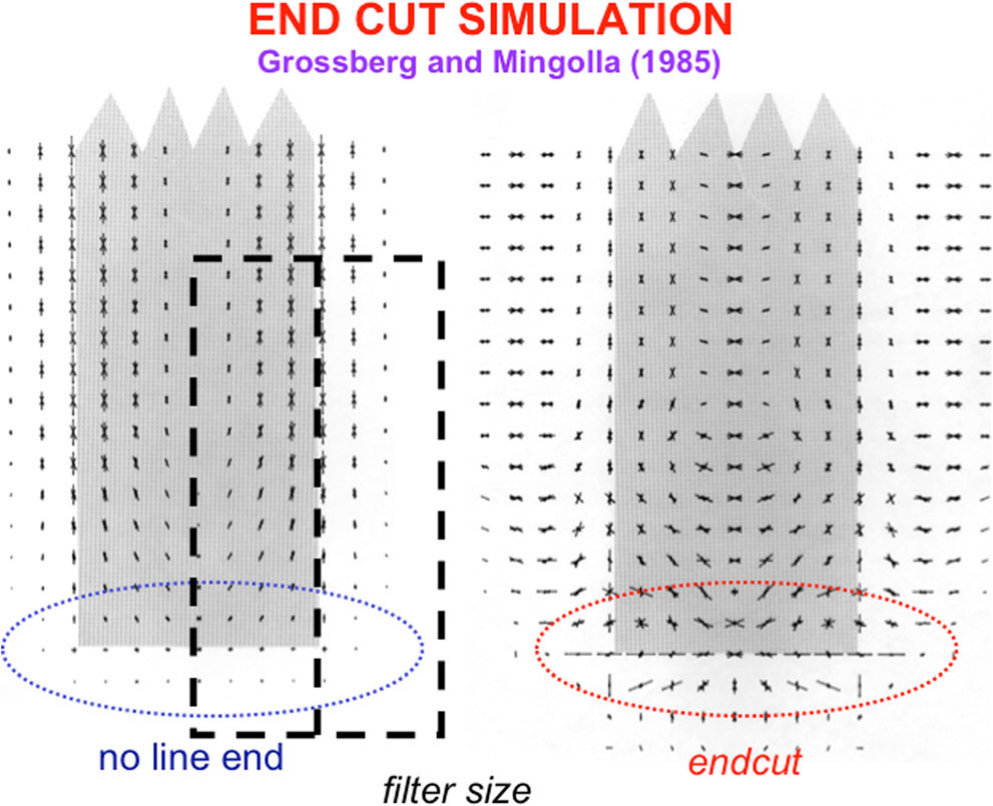
The left image shows a computer simulation of the responses of model complex cells to a line end. The line end is shown in gray. The spatial scale of the cells is shown by the dark hatched double-rectangular region. The magnitude of an oriented cell’s response at each position is proportional to the length of the line drawn with the same orientation at that position. Note that, although strong vertical and nearly vertical responses occur along and near the sides of the line, there are no responses at the line end. The right image shows the end cut that is created at the hypercomplex cells by inputs from these complex cell responses. Note that the end cut is positionally hyperacute but orientationally fuzzy. The latter property enables end cuts to form parts of groupings that are perpendicular and nearly perpendicular to line ends, as in the percept of neon color spreading in Fig. 4. Adapted with permission from Grossberg and Mingolla (1985).(Color figure online)

Spatial and orientational competition close boundary gaps at line ends using end cuts (see Figs. 8 and 9; Grossberg & Mingolla, 1985). The simple, complex, and hypercomplex cells in Fig. 5 thus illustrate a hierarchical resolution of uncertainty that overcomes the spatial uncertainty at line ends that is caused by using simple cell receptive fields.

Although end cuts do not prevent all spreading of brightness and color from occurring, as neon color spreading illustrates (see Fig. 4), events like neon color spreading are rare. They also provide useful evidence for how our brains form boundaries and surfaces by showing how these processes can occasionally break down.

### Complex cells can detect boundaries where contrast polarities reverse, but cannot see qualia

**Fig. 10:**
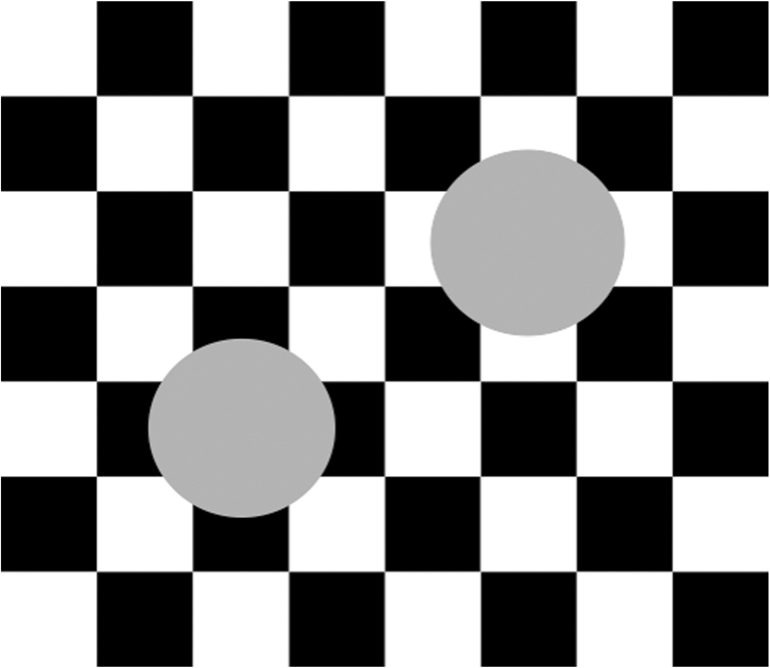
As the circumferences of the two disks are traversed, the relative contrast reverses periodically, from dark-to-light (gray-to-white) to light-to-dark (gray-to-black) and back again. Although simple cells that are sensitive to one contrast polarity could not form a boundary around the entire circumference of these disks, hypercomplex cells can. This image also creates a strong amodal percept that the upper right disk occludes a white cross, whereas the lower left disk occludes a black cross. These percepts violate a Bayesian account of the most probable percept that an occluded checkerboard lies behind these gray disks

Before output signals from simple cells reach the hypercomplex cells, they overcome a different kind of uncertainty. Each simple cell can respond to either a light-dark or a dark-light oriented contrast within its receptive field, but not to both (see Fig. 6). If simple cells that are sensitive to just one contrast polarity, say light–dark, input to the competitive stages, then the brain would often create boundaries with big gaps in them. This would occur, for example, in response to objects that lie in front of textured backgrounds whose relative contrasts with respect to the background reverse along the object’s perimeter (see Fig. 10). Brightnesses and colors could spread out of these boundary gaps as well, again obliterating critical scenic information. This perceptual catastrophe is averted by using complex cells at which the outputs of like-oriented dark–light and light–dark simple cells are added at each position (see Fig. 6; Hubel & Wiesel, 1968). Complex cells can then respond to contrasts of both polarities at *every* position along the bounding contour of an object in front of a textured background.

Pooling inputs from opposite contrast polarities at complex cells implies that boundaries cannot represent visual qualia. They cannot discriminate between dark–light and light–dark contrasts, or red–green and green–red contrasts, or blue–yellow and yellow–blue contrasts, because they pool together inputs from simple cells that are sensitive to all of these differences (Thorell, de Valois, & Albrecht, 1984) to form the best possible boundaries. In other words, boundaries are *insensitive to direction of contrast*. Although boundaries can vary in strength or distinctiveness as they receive inputs from variable numbers and strengths of inducers, they do not code for visible brightnesses or colors.

**Fig. 11:**
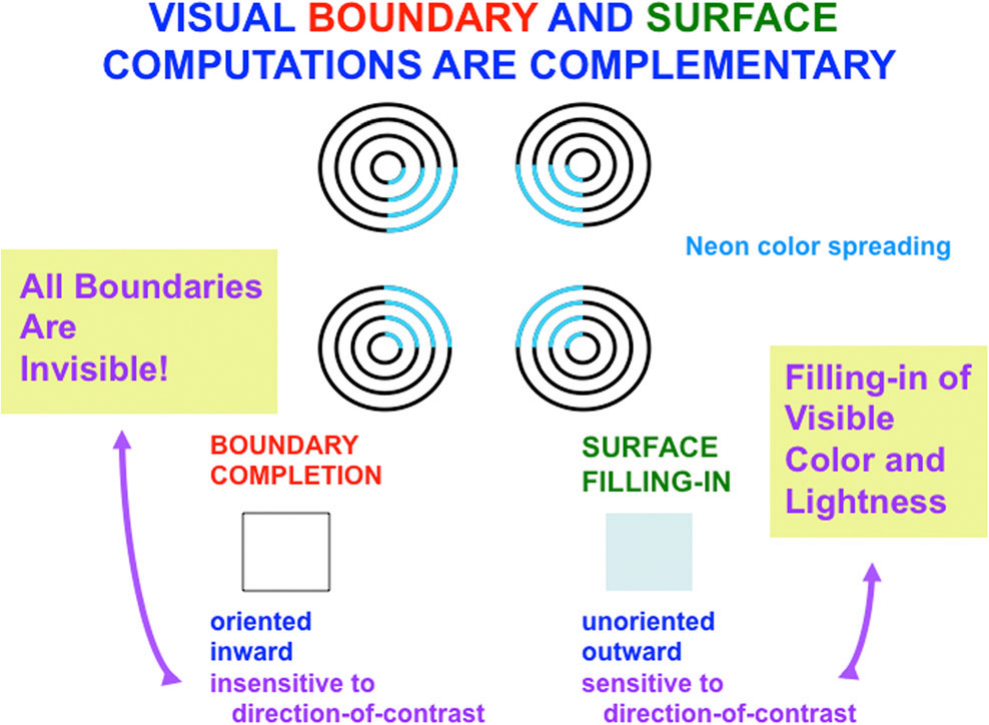
Neon color spreading illustrates computationally complementary properties of boundary completion and surface filling in, notably that all boundaries are invisible, and that visible qualia are surface percepts. (Color figure online)

**Fig. 12:**
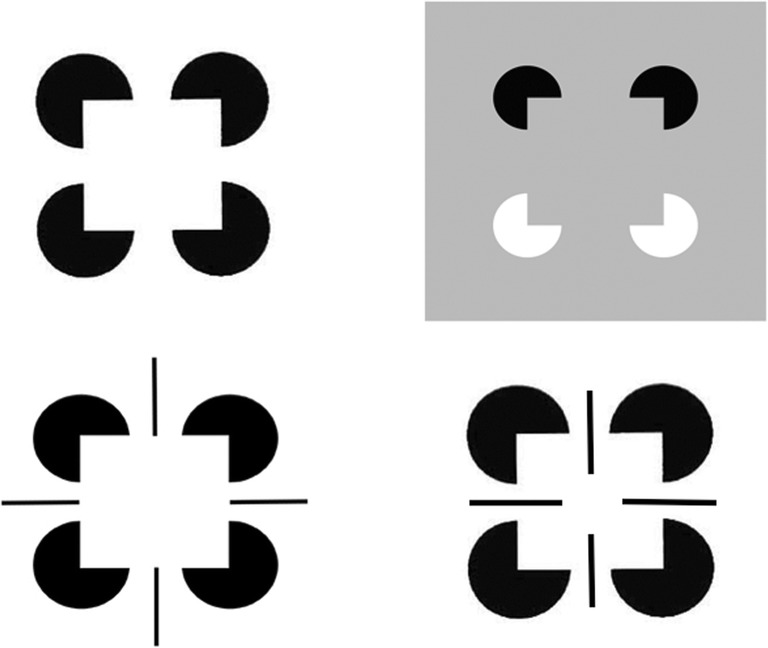
The image in the upper row (left) is a Kanizsa square. The illusory square looks brighter than its background and in front of four partially occluded disks whose unoccluded parts have the shape of Pac-Man figures. See Grossberg (2014) for an explanation of how the apparent brightness and depth of the emergent square covary. The image in the upper row (right) is a reverse-contrast Kanizsa square. The illusory square can be recognized, but many people do not see it because the filled-in gray colors inside and outside the square are approximately equal. This is due to the fact that there are two white Pac-Men and two black Pac-Men on a gray background. The white Pac-Men cause darker feature contours within the illusory square, whereas the black Pac-Men cause brighter feature contours within the illusory square. When these darker and brighter feature contours fill in within the square, they tend to cancel out. The same thing happens outside the square. The net effect is a similar gray color both inside and outside the square. The square thus can be recognized, but not seen. In the lower row, two Kanizsa square percepts are generated using additional lines that either abut the emergent square boundary or penetrate it, leading to dramatically different percepts. (Color figure online)

Said in a more paradoxical way: *All boundaries are invisible* (see Fig. 11). Boundaries may be consciously recognized, even when they are invisible, as in the boundaries formed by the reverse-contrast Kanizsa square image in Fig. 12 (top row, right column).

If boundaries are invisible, then how do we see anything? *Consciously perceived qualia are predicted to be surface percepts* (see Fig. 11). Visible surface percepts can be generated by different surface brightnesses or colors that may occur on two sides of a boundary after surface filling-in occurs, as illustrated by the enhanced brightness of the squares generated by the Kanizsa square stimuli in Fig. 12 (top row, left column; bottom row).

### Boundary completion closes retinal boundary gaps using bipole grouping cells

Many boundaries would still remain incomplete if boundary processing stopped with hypercomplex cells. For example, the two Kanizsa squares in the top row of Fig. 12 would just be seen and recognized as four Pac-Man figures. Why does the brain bother completing boundaries, indeed *illusory* boundaries, between pairs of colinear Pac-Man edges?

**Fig. 13:**
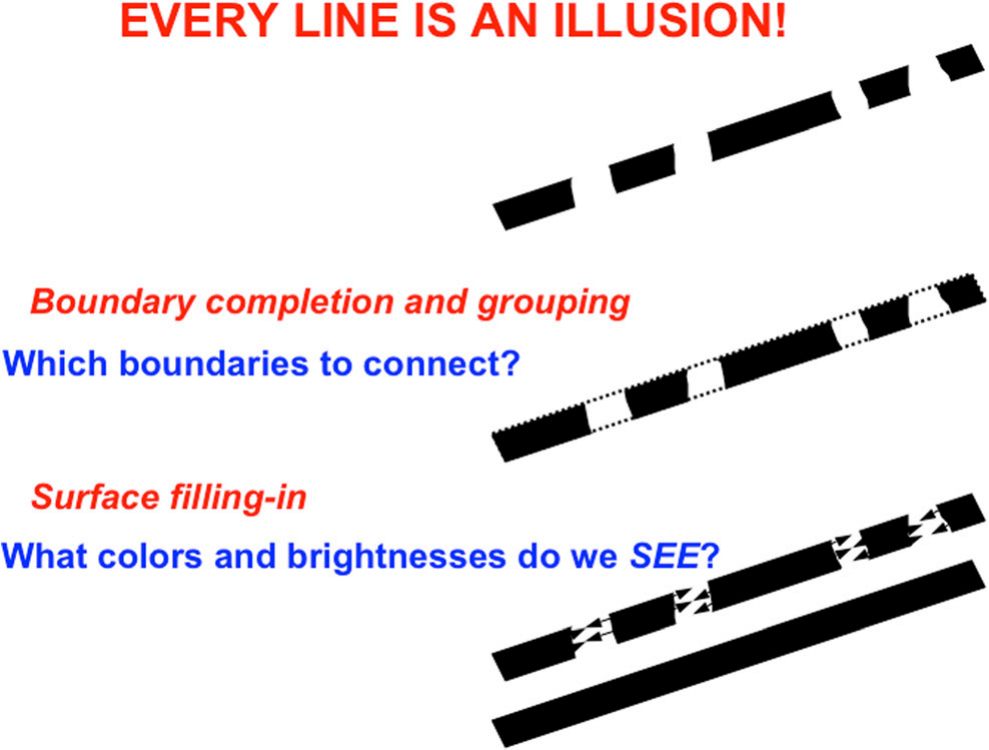
Even the straight line in Fig. 2 can be occluded in multiple positions by the blind spot and retinal veins (top image). To complete the occluded representation in the top image of this figure, both boundary completion (middle image) and surface filling-in (bottom image) are needed

There are several important functional reasons for doing this. One reason is that the retinal blind spot and veins prevent the processing of connected objects that are registered by the retina at their positions (see Fig. 2). The process of boundary completion generates boundaries of these objects across the blind spot and retinal veins, as well as the boundaries of Kanizsa squares (see Fig. 13).

**Fig. 14:**
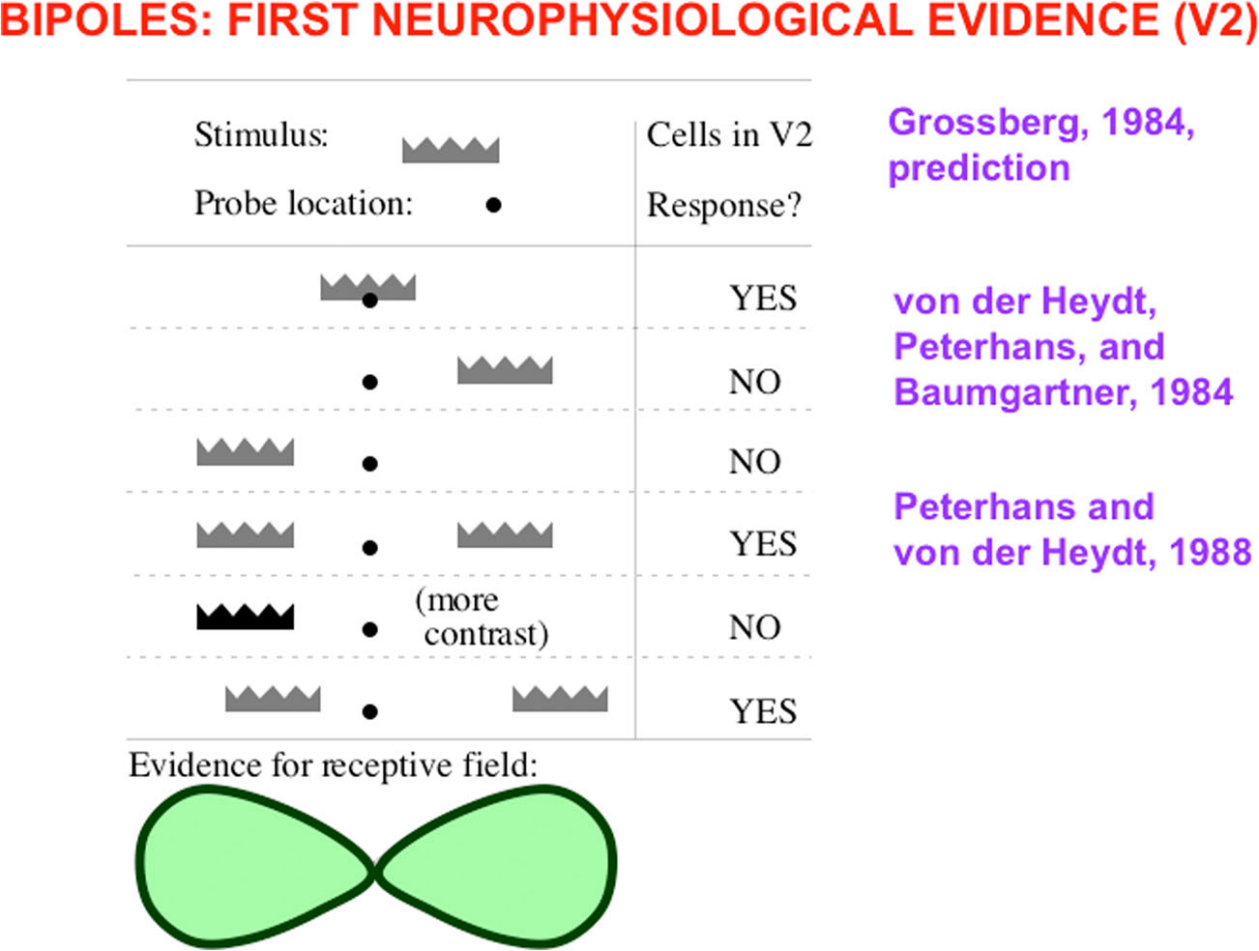
Bipole cell properties in cortical area V2 were first reported by von der Heydt, Peterhans and Baumgartner (1984). The various cases of cell response and nonresponse, as recorded at the probe location, clarify that, either direct activation of the cell at the probe location, or approximately like-oriented input stimuli to both branches, or “poles,” of the cell’s receptive field, are needed to fire the cell, and this continues to be true if the positions of these oriented inputs are moved back and forth within these branches

Boundary completion cells receive their inputs from hypercomplex cells (see “To cooperation” in Fig. 5). They then cooperate across space with other boundary cells to complete a boundary between them whenever these cells are near enough to one another, are aligned across space in an approximately collinear arrangement, and have orientational tuning that is also approximately aligned. These boundary completion cells are often called *bipole cells* (see Fig. 14; Cohen & Grossberg, 1984; Grossberg, 1984; Grossberg & Mingolla, 1985) because they complete boundaries *inwardly* in an *oriented* manner between pairs (bipoles!) of boundary inducers. Predicted bipole grouping properties have been supported by psychophysical data (e.g., Field, Hayes, & Hess, 1993; Kellman & Shipley, 1991), and neurophysiological data from cells in cortical area V2 (e.g., Peterhans & von der Heydt, 1989; von der Heydt, Peterhans, & Baumgartner, 1984). Variants of bipole cells have also been used by other authors to model boundary grouping (e.g., Heitger & von der Heydt, 1993; Williams & Jacobs, 1997).

**Fig. 15:**
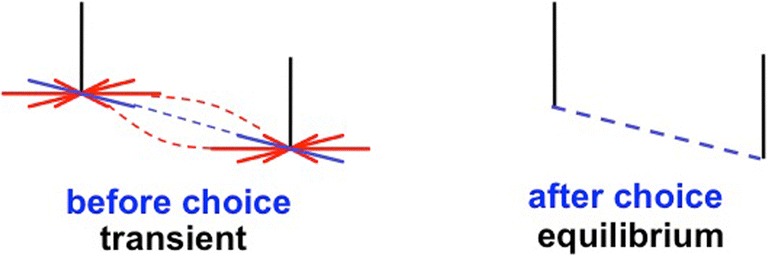
(Left panel) The bipole cell receptive field enables multiple nearby orientations and positions to initiate grouping. (Right panel) Despite this initially coarse grouping, the final grouping is often sharp in both its positional and orientational selectivity due to feedback interactions within the entire network

When a bipole grouping starts to form, it is often initially fuzzy across space (see Fig. 15, left image). If perfect alignment of inducers were required before grouping could start, then there would be a vanishingly small probability that boundary completion could begin. Instead, bipole cell receptive fields are coarse enough to enable multiple nearly collinear and nearly orientationally aligned inducers to start the grouping process. This coarseness embodies within bipole receptive fields the many perceptual experiences with nearly collinear and aligned visual stimuli during cortical development (Grossberg & Swaminathan, 2004; Grossberg & Williamson, 2001). However, if all perceptual groupings remained fuzzy, visual acuity would be degraded. Instead, feedback within the boundary system can rapidly choose a final sharp grouping that is maximally consistent with the spatial organization of the positional and orientational evidence in all of its inducers (see Fig. 15, right image).

To summarize what has already been described: The first hierarchical resolution of uncertainty uses hypercomplex cells to complete boundaries at line ends and corners that simple cells cannot detect. The second hierarchical resolution of uncertainty uses bipole grouping to complete positionally sharp boundaries at positions that are occluded by the blind spot and retinal veins, or behind occluders in any scene or image. The third hierarchical resolution of uncertainty concerns why surface filling-in occurs.

### Filling-in completes surface representations after the illuminant is discounted

Completed boundaries input topographically to surface representations where they are both generators of, and barriers to, surface filling-in (Grossberg, 1994, 1997; Grossberg & Yazdanbakhsh, 2005; Kelly & Grossberg, 2000). These boundary-to-surface signals are predicted to occur from boundary representations within the interstripes of cortical area V2 to surface representations within Filling-In DOmains, or FIDOs, of the thin stripes of cortical area V2 (Figure 3). Each FIDO also receives bottom-up topographic brightness or color signals. For example, when blue color inputs in response to the neon color image in Fig. 4 activate the corresponding FIDO, blue color can spread *outward* in an *unoriented* manner across this FIDO. In particular, because the boundaries of the blue lines in Fig. 4 have lower contrast than those of the black lines, end gaps form in the boundaries generated by the blue lines where they abut the black lines. Blue color can flow out of these end gaps and spread across space until it hits the square illusory boundary that is completed by bipole grouping, which prevents its further spread.

In addition to its outward and unoriented spread, surface filling-in is also *sensitive to direction of contrast*, because we can consciously see its effects. Neon color spreading hereby illustrates three pairs of computationally complementary properties of boundary completion and surface filling-in (see Fig. 11): oriented versus unoriented; inward versus outward; insensitive to direction of contrast versus sensitive to direction of contrast. A good boundary completion process thus cannot also be a good surface filling-in process, and conversely. Interactions between these processes overcome their complementary deficiencies to generate completed boundaries and filled-in surfaces.

In what sense is surface filling-in an example of hierarchical resolution of uncertainty? The surface system “discounts the illuminant,” or compensates for variable illumination, at an early processing stage. If this did not happen, then the brain could erroneously process changes in illumination as changes in perceived object shapes and colors. If object shapes could plastically deform whenever illumination changed, then the brain could not learn to recognize a stable object percept.

**Fig. 16:**
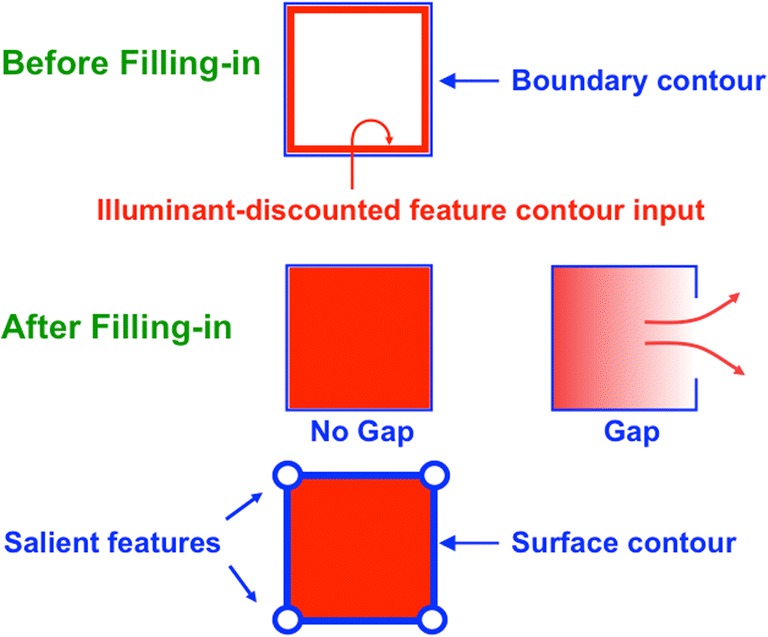
(Top row) A closed boundary contour (in blue) surrounds a pattern of illuminant-discounted feature contour activities (in red) before filling-in occurs. (Middle row, left column) After filling-in within the closed boundary, the filled-in activity spreads through the entire surface region within the rectangular closed boundary. (Middle row, right column) If there is a large hole, or gap, in a boundary, then color can flow out of it and equalize the filled-in surface activity on both sides of the boundary. Because of this difference, closed and open boundaries are processed differently during figure–ground separation. (Bottom row) Output signals form along the bounding contour of a filled-in surface that is surrounded by a closed boundary. The activity of cells in this surface contour is greater at the positions of salient features like the corners of the rectangle. (Color figure online)

The illuminant-discounting process inhibits luminance and color inputs at many positions across a scene’s surface. The process spares signals, called *feature contours*, near positions where color or luminance changes sufficiently rapidly across space. The feature contours that survive discounting of the illuminant in response to a square red rectangle are shown at the top of Fig. 16 in the image that is labeled Before Filling-in. Feature contours are computed at positions where material properties of scenic objects change, not just their illumination. At these positions, feature contours can compute material properties of a surface, such as its *reflectances*, or the proportions of reflected light within each wavelength. These reflectances are insensitive to illumination changes. In Fig. 16, the feature contours form a rectangular red region just inside the closed boundary contour (in blue) that surrounds the rectangle.

At a later FIDO processing stage, surface filling-in spreads feature contour brightnesses and colors within the closed rectangular boundary contours to reconstruct a surface representation of the scene in which the illuminant is significantly discounted. This filled-in red rectangle is depicted in the After Filling-in image that is directly under the feature contour figure, above the label No Gap. Henceforth, this filled-in figure will be said to occur in Fig. 16 (left column, middle row). Surface filling-in of illumination-discounted feature contours is the third hierarchical resolution of uncertainty.

## 8. Recognizing occluded objects while seeing unoccluded opaque surfaces and transparent ones

The above boundary and surface interactions are necessary to understand how conscious states control actions, but they are not sufficient. In order to make the links to consciousness and action, it is necessary to also understand how boundaries and surfaces support 3-D figure–ground separation. The above properties of boundaries and surfaces have considered only how they work in two dimensions, or 2-D. In the real world, however, boundary completion and surface filling-in do their work in response to 3-D scenes that may contain partially occluded objects. In a 3-D world, the following questions also need to be answered: How do we recognize completed objects behind their occluders? Why do we only see the unoccluded parts of opaque objects, yet can also see occluded objects behind transparent occluders? How do conscious states respond to such figure–ground representations to trigger actions?

**Fig. 17:**
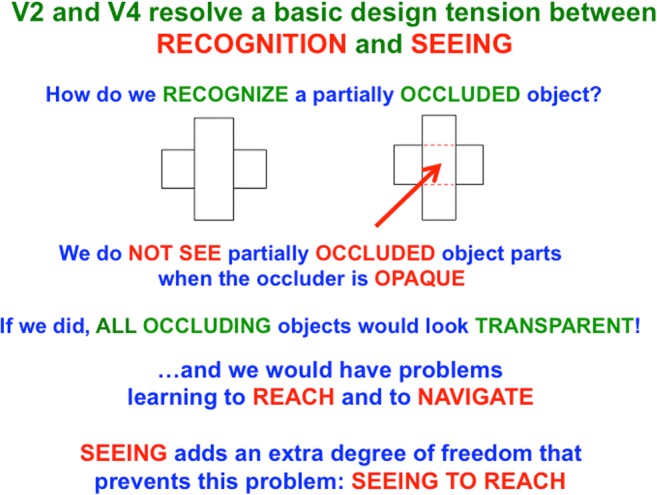
(Left column) Three abutting rectangles cause a compelling percept of a vertical bar that partially occludes a horizontal bar. (Right column) The occluded region of the horizontal bar is amodally recognized without being consciously seen. If all such completed occluded regions could be seen, then all occluders would look transparent. Interactions between cortical areas V2 and V4 are predicted to prevent this from happening. See the text for details about how this is proposed to happen

**Fig. 18:**
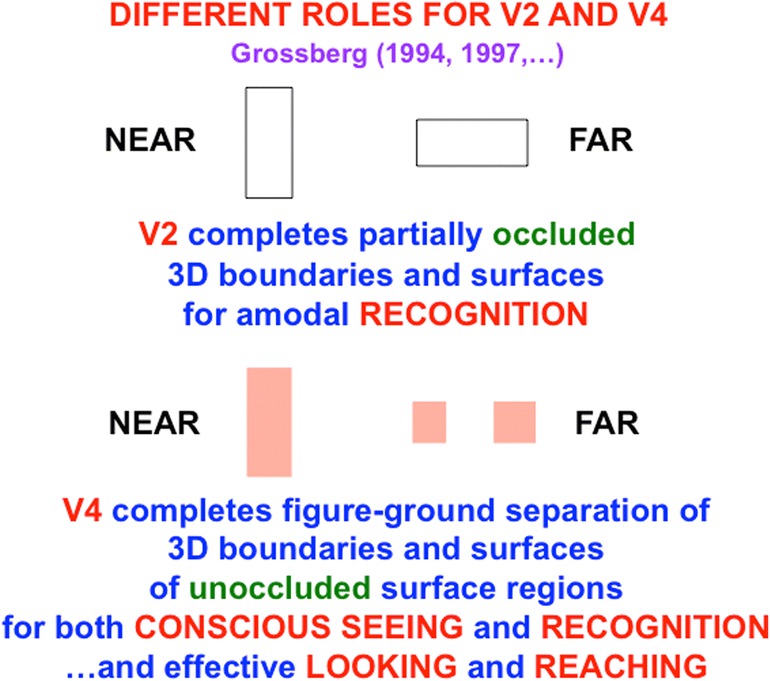
Whereas cortical area V2 is predicted to complete depth-selective amodal boundary and surface representations of the bars in Fig. 17 for purposes of recognition, cortical area V4 is predicted to fill-in depth-selective unoccluded surface regions for conscious seeing and recognition, and for looking and reaching

The percept that is generated by the left image in Fig. 17 illustrates this issue. Although this image is composed of three abutting rectangles, it generates a compelling percept of a horizontal bar that is partially occluded by a vertical bar. The partially occluded portion of the horizontal bar (see Fig. 17, right image) is recognized, but it is not consciously seen. The FACADE model of 3-D vision and figure–ground perception proposes how this vertical rectangle is separated from the partially occluded horizontal rectangle, including the boundaries which they share, after which the horizontal rectangle can be completed “behind” the horizontal rectangle (see Fig. 18; Fang & Grossberg, 2009; Grossberg, 1994, 1997; Grossberg & Swaminathan, 2004; Grossberg & Yazdanbakhsh, 2005; Kelly & Grossberg, 2000). More will be said about how this is proposed to happen below, and in Section 12.

### Closed boundaries contain depth-selective filling-in

How did evolution discover figure–ground separation? Remarkably, properties of figure–ground separation emerge from interactions that compute *complementary consistency*. Recall from Figs. 4 and 11 that the rules that govern boundary completion and surface filling-in are computationally complementary. Nonetheless, we typically consciously see, with fixed attention, only one percept in response to an image, except in special circumstances such as those that cause binocular rivalry. Complementary consistency is realized when the signals within V2 from the boundary stream to the surface stream that create barriers to the filling-in of object surfaces trigger feedback signals from the surface stream back to the boundary stream. To understand how this works, consider the image in Fig. 16, labeled Gap (right column, middle row). This image has a big gap, or hole, in its boundary. As a result, brightness and color can flow out of the boundary into the surrounding image, and conversely. The net effect is to equalize brightness and color contrasts on both sides of the boundary.

### Surface contours are activated at positions where closed boundaries contain filling-in

The processing stage after surface filling-in occurs computes topographically organized feedback signals that are called *surface contours* back to its generative boundaries. Surface contours are generated by contrast-sensitive on-center off-surround networks that act across space and within each depth. Because of their contrast sensitivity, these networks generate output signals only at positions where they detect a rapid change in contrast across space. Such rapid contrast changes occur only at the contours of successfully filled-in surfaces, which are the surfaces that are surrounded by closed boundaries. Such a filled-in surface has already been described by the No Gap image in Fig. 16 (middle row, left column). The surface contour that is generated by this filled-in surface is shown just below it as a blue rectangle around the red region in Fig. 16 (bottom row, left column). The open blue circles at the corners of the blue rectangle are positions of enhanced surface contour activity whose cause, and function, will be explained below.

Surface contours are *not* generated at positions where open boundaries occur, as in response to the boundary Gap image in Fig. 16 (middle row, right column) because the surface filling-in that is caused by feature contours within regions with open boundaries can spread to both sides of their boundaries, and thus do not generate large contrasts at boundary positions.

### Surface contours realize complementary consistency and initiate figure–ground separation

Surface contours can support both complementary consistency and figure–ground separation using the property, shown in Fig. 16, that surface contours form around filled-in surfaces that are surrounded by closed boundaries, but not around surfaces whose color and brightness can flow out of big boundary gaps. How this property helps to realize complementary consistency is clarified by da Vinci stereopsis percepts of 3-D scenes that each eye can see to different degrees. This often occurs during viewing of objects in natural scenes when a nearer object occludes part of the surface of a farther object (Cao & Grossberg, 2005, 2012; Nakayama & Shimojo, 1990).

**Fig. 19:**
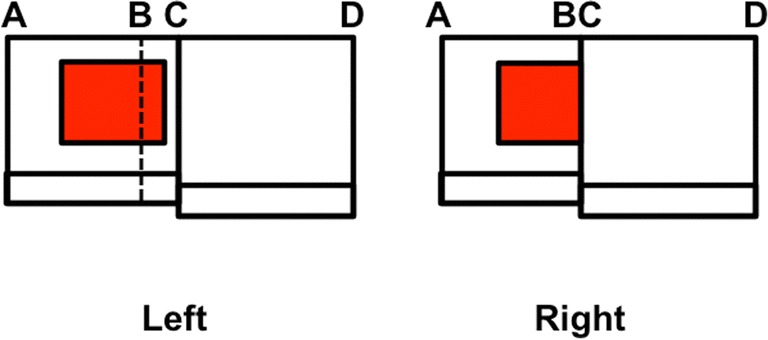
An example of a simple scene that illustrates da Vinci stereopsis, as seen through the left (L) and right (R) eyes in depth. See the text for details

Figure 19 illustrates how this can happen. In this scene, one wall, between the edges C and D, is closer to the observer than the other wall. The observer’s left eye sees more of the wall on which the rectangular red picture hangs. In particular, only the left eye can see the wall in the region between positions B and C. In the right eye view, part of the picture is occluded by the nearer wall, which is why the positions B and C are identified. Remarkably, the consciously perceived depth of the monocularly perceived surface between B and C is derived from the binocularly perceived depth of the surface between A and B.

In order to fill in the red picture at this farther depth, the brain first needs to create a closed boundary around it at this depth. However, only the left vertical boundary of the red picture is binocularly seen. How do the picture’s other three boundaries get created? In particular, how does the brain decide to what depth, or depths, the other boundaries, which do not generate strong depth signals, should be assigned? Grossberg (1994) predicted that such boundaries are assigned to *all* depths along their lines of sight in the V2 interstripes where binocular boundaries are computed (see Fig. 3). Yazdanbakhsh and Watanabe (2004) published psychophysical experiments that support this prediction by showing an “asymmetry between horizontal and vertical illusory lines in determining the depth of their embedded surface” (p. 2621).

**Fig. 20:**
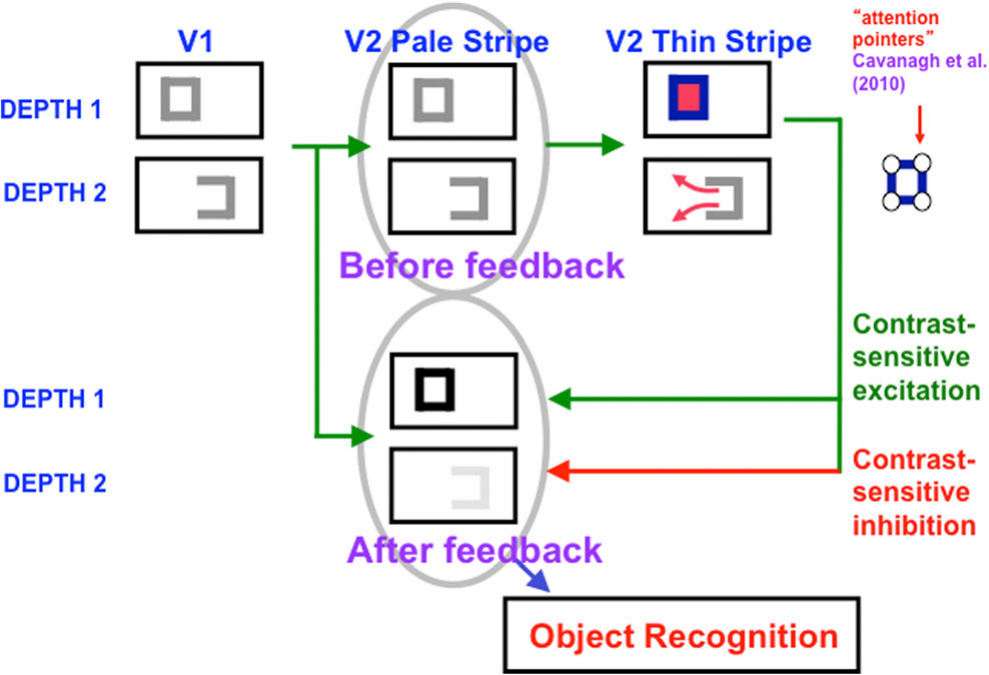
Surface contour signals ensure complementary consistency of boundaries and surfaces while also initiating figure–ground separation. The filled-in surface color or brightness of a region that is surrounded by a closed boundary (at Depth 1 of V2 thin stripes) can generate surface contour signals at the positions of that boundary. These surface contour signals strengthen the boundaries that induced the surface (at Depth 1 of V2 pale stripes), while inhibiting the spurious boundary signals at the same positions, but further depths (at Depth 2 of V2 pale stripes). With these spurious boundaries eliminated, partially occluded objects (not shown) can be amodally completed at the further depth plane (Depth 2) via collinear bipole boundary completion. A percept of two figures, one partially occluding another, can thereby be generated, as in response to the image in Figure 17 (left column)

Assume for definiteness that the binocular disparity of the left vertical boundary of the red picture in Fig. 19 assigns it to Depth 1 in Fig. 20, where it is displayed as the left vertical boundary in both V1 and in V2 pale stripes. The remaining boundaries, which form a reversed C shape in Fig. 20, are projected along their line of sight to all the depths. Figure 20 depicts only two depths, Depth 1 and Depth 2, for simplicity, but the argument generalizes to an arbitrary finite number of depth planes. In the V2 pale stripes of Fig. 20 (top row, middle column), this projection creates a closed boundary only at Depth 1, and open boundaries at all other depths.

These completed boundaries are topographically projected to the V2 thin stripes, where they control surface filling-in. Figure 20 (top row, right column) shows that only the closed rectangular boundary at Depth 1 can contain the filling-in of the picture’s red color. The open boundary at Depth 2 allows color to spread to both of its sides, as in the Gap image of Fig. 16. As a result, surface contours form only at Depth 1 at the same positions where boundaries, acting as filling-in barriers, block the spread of the filling-in process (cf. Fig. 16, middle row, left column). This rectangular surface contour is depicted in the V2 thin stripes of Fig. 20 as a blue rectangle.

As shown in Fig. 20, these surface contours deliver topographic feedback signals to the boundary representations that generated them. This is the feedback process that achieves complementary consistency. It is accomplished by an on-center off-surround network that is depicted by a downward-and-leftward green arrow (labeled contrast-sensitive excitation) and a leftward red arrow (labeled contrast-sensitive inhibition) in row two of Fig. 20. The on-center signals strengthen the boundaries that generated the successfully filled-in surface. This strengthened boundary at Depth 1 is depicted as a black rectangle in Fig. 20 (middle column, second row) in the V2 pale stripe representation that is labeled After Feedback. The inhibitory connections in the off-surround act *within position* and *across depth* and thereby inhibit redundant boundaries at the same positions but farther depths. This inhibitory process is called *boundary pruning*. The inhibited inverted C boundary at Depth 2 is depicted in light gray in Fig. 20 (middle column, second row).

This off-surround network from the nearer Depth 1 to the farther Depth 2 is an example of the *asymmetry between near and far*, which develops from experience because, among other things, we can walk forward but not backward, at least most of the time.

Complementary consistency is hereby realized by confirming and strengthening the boundaries that lead to successful surface filling-in while inhibiting those that do not.

Figure 20 also indicates how complementary consistency enables figure–ground separation to begin: By eliminating all redundant boundaries of an occluding object at farther depths (e.g., Depth 2), collinear boundaries that abut the occluding object at these depths can be amodally completed behind it, as in Fig. 17. Figure 20 does not explain all that has to happen for figure–ground separation to be completed. One also needs to explain how, in response to an image like the three abutting rectangles in Fig. 17, the vertical boundaries where the smaller rectangles touch the vertical occluding rectangle “belong” to the occluding rectangle, while being detached from the smaller rectangles, and how this event drives the representations of both smaller abutting rectangles to a further depth plane, in this case, Depth 2. After that happens, due to the boundary pruning shown in Fig. 20, their occluded boundaries can be collinearly completed behind the vertical occluding rectangle, as in Fig. 18 (top row, right column).

The FACADE model explains how this boundary separation and completion process occurs in cortical area V2, and uses the same bipole cells that complete boundaries of objects that are occluded by the retinal veins and blind spot (see Figs. 13 and 14). Then, direct pathways from V2 to higher cortical areas such as inferotemporal (IT) cortex, and back, are used to *recognize* this completed perceptual representation as part of a *feature–category resonance* (see Fig. 1), despite the fact that the occluded part of this rectangle is not consciously *seen*. Such recognition without seeing is said to be *amodal*.

### Why do not all occluders look transparent?

If the completed boundary and surface behind the vertical rectangle could also be seen, then the vertical rectangle would look transparent, because both the horizontal rectangle, and the vertical rectangle in front of it, could be seen at the same spatial positions. If the completed parts of partially occluded objects could always be seen, then *all occluders would look transparent*. Confusion could then occur in the planning of looking and reaching behaviors because it could seem natural to reach directly through occluding objects to the occluded objects behind them. There is thus a *design tension during evolution between the requirements of recognition and reaching*. Conscious visibility enables the unoccluded parts of many surfaces to appear opaque, and thus good targets for reaching, without eliminating the ability of the visual cortex to correctly represent surfaces that are, in fact, transparent.

### Completed V2 occluded regions are amodal, whereas unoccluded V4 regions are visible

The FACADE model predicts how cortical areas V2 and V4 work together to ensure that not all occluding objects look transparent: Cortical area V2 is proposed to complete object boundaries and surfaces of *occluded* object regions that may be amodally recognized, but not seen. Animals who could not recognize such partially occluded objects, such as a predator that is partially occluded by vegetation, would be at a severe survival disadvantage compared with those who could. Cortical area V4 is predicted to be the cortical region where figure–ground-separated 3-D surface representations of the *unoccluded* regions of *opaque* object regions are completed, and thereupon support both seeing *and* recognition of these regions (see Fig. 18). These unoccluded object surface regions are the parts of a scene that are typically consciously seen as we explore the world, and are used to control looking and reaching movements. The same model neural mechanisms also explain how V4 also supports seeing of 3-D surfaces that really are transparent (Grossberg & Yazdanbakhsh, 2005).

The hypothesis that V4 represents 3-D surfaces whose objects have been separated from one another in depth is consistent with several different types of neurobiological experiments (e.g., Chelazzi, Miller, Duncan, & Desimone, 2001; Desimone & Schein, 1987; Lueck et al., 1989; Ogawa & Komatsu, 2004; Reynolds, Pasternak, & Desimone, 2000; Schiller & Lee, 1991; Zeki, 1983). Additional experiments that distinguish between recognizing and seeing occluding and occluded objects regions are much to be desired.

## 9. Surface–shroud resonances between V4 and PPC control conscious seeing and action

A surface–shroud resonance (see Table 1a) is thus assumed to be triggered between V4 and PPC because V4 is predicted to be the cortical stage at which figure–ground-separated 3-D surface representations of unoccluded surface regions are computed. Such a surface–shroud resonance provides a conscious surface visibility signal to mark the opaque unoccluded surface regions to which orienting eye movements and reaching arm movements can be successfully directed.

**Fig. 21:**
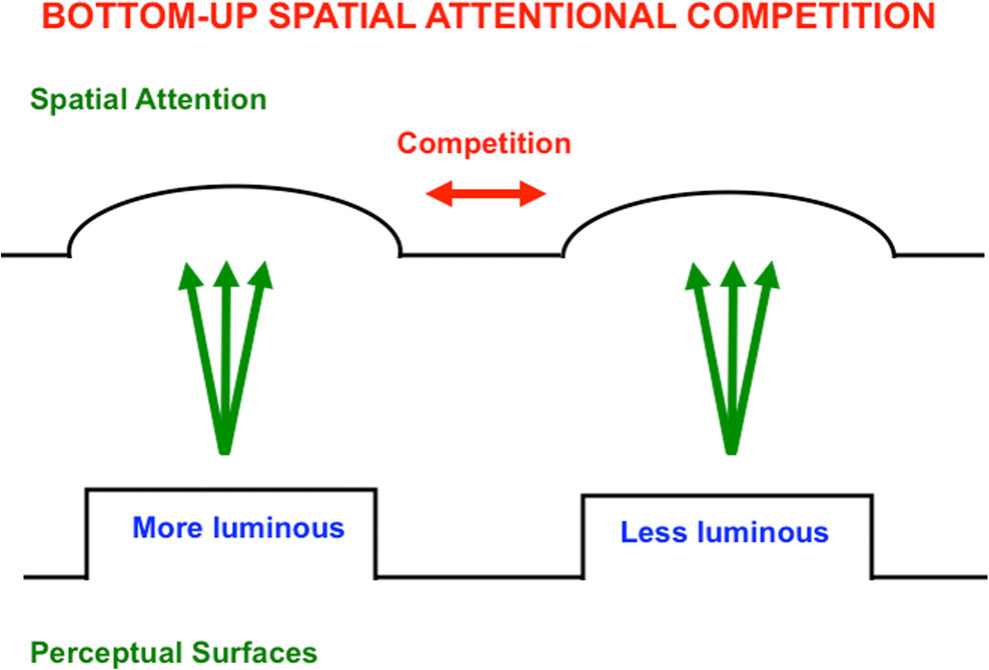
A cross-section of a simple filled-in surface (e.g., in cortical area V4) is shown in which a more contrastive bar is to the left of a less contrastive bar. Each position in the surface sends topographic bottom-up excitatory signals to the spatial attention region (e.g., PPC) where the activated cells compete

**Fig. 22:**
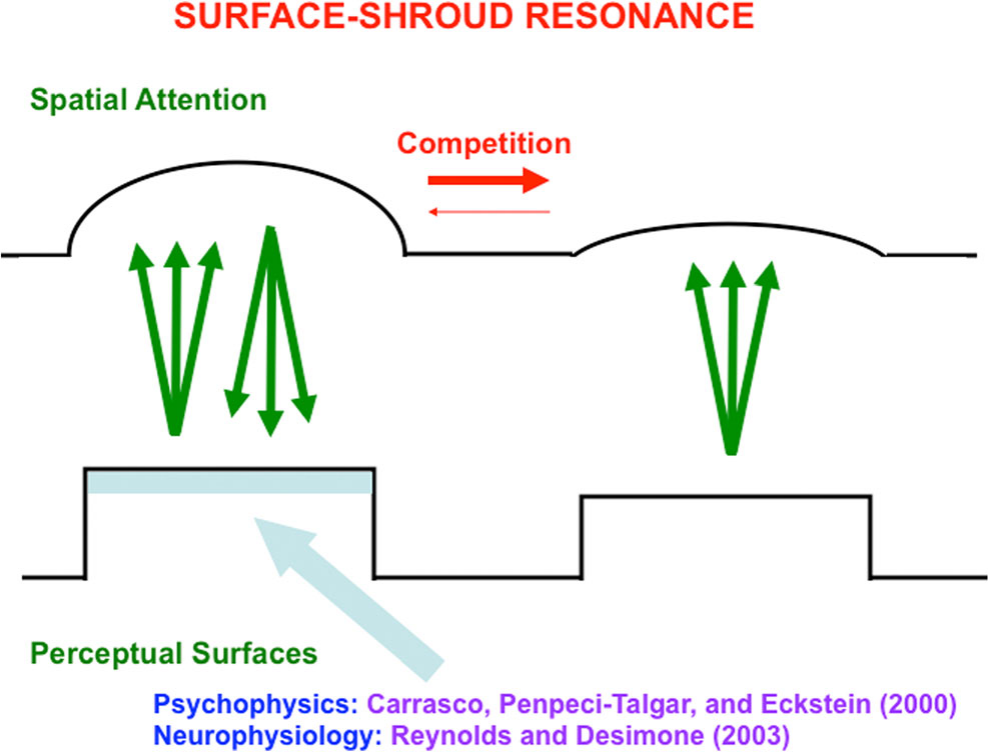
Each activated spatial attention cell sends topographic top-down excitatory signals to the corresponding surface, while it also sends broad off-surround inhibitory signals to other spatial attention cells, thereby activating a recurrent on-center off-surround network whose cells obey shunting laws. This recurrent network generates a surface–shroud resonance that contrast-enhances the more active spatial attention cells, while inhibiting the less active ones, thereby creating a form-sensitive distribution of spatial attention, or attentional shroud, that focuses spatial attention upon the more contrastive surface, while also increasing its effective contrast

Figures 21 and 22 summarize how a surface–shroud resonance forms. Figure 21 depicts a one-dimensional cross-sectional area of a simple surface representation in V4 that consists of two bars, one with a higher luminance than the other. This surface representation sends topographic excitatory signals to the spatial attention region in PPC, where the activations that they cause begin to compete across space. As this is going on, top-down excitatory feedback signals are generated by the activated spatial attention cells back to their inducing surface representations (see Fig. 22). These recurrent excitatory and inhibitory signals form a recurrent on-center off-surround network of interactions between neurons that obey the membrane equations of neurophysiology, also called shunting interactions. Such a network contrast-enhances its largest inputs while suppressing smaller inputs and approximately normalizing its total activity (Grossberg, 1973, 1980). A surface–shroud resonance is the result, with form-fitting spatial attention constituting the attentional shroud, and enhancement of perceived brightness of the attended surface by the shroud’s top-down excitatory feedback signals, an enhancement that has been reported both psychophysically (e.g., Carrasco, Penpeci-Talgar, & Eckstein, 2000) and neurophysiologically (e.g., Reynolds & Desimone, 2003).

## 10. Feature–category resonances for recognition and surface–shroud resonances for seeing

**Fig. 23:**
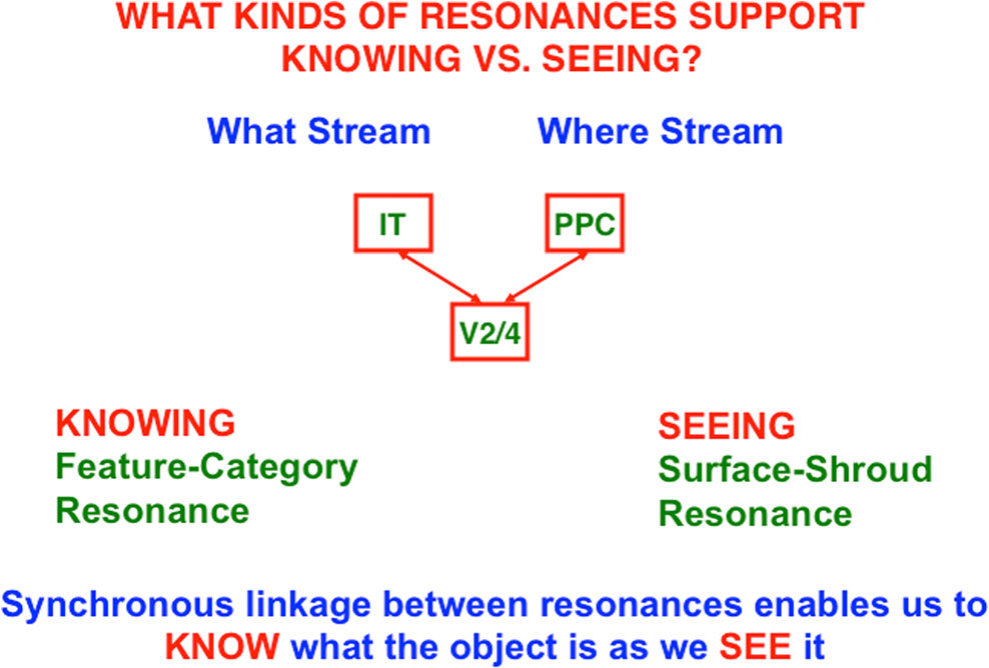
Seeing and knowing. A surface–shroud resonance that supports conscious seeing and a feature–category resonance that supports conscious knowing, or recognition, can occur simultaneously and be supported by a synchronous resonance that bridges the “what” and “where” cortical streams via shared prestriate visual cortical circuits

The fact that invisible boundaries can be recognized and that all conscious qualia are surface percepts suggests that boundaries and surfaces both contribute to resonances that support recognition and/or seeing. Often when we consciously *see* a familiar object, we also *know* what it is. ART proposes that these two kinds of awareness are due to different kinds of resonances (see Fig. 23), with knowing, or recognizing, supported by feature–category resonances that include “what” stream regions such as IT, and seeing supported by surface–shroud resonances that include “where/how” stream regions such as PPC. We know what a familiar object is when we see it because both resonances interact with shared visual cortical areas, such as V2 and V4, and can thus synchronize with each other, often with gamma oscillations (cf. Fries, 2009; Gregoriou, Gotts, Zhou, & Desimone, 2009; Grossberg & Versace, 2008; Lamme, 2006; Llinas, Ribary, Contreras, & Pedroarena, 1998; Pollen, 1999; Singer, 1998).

**Fig. 24:**
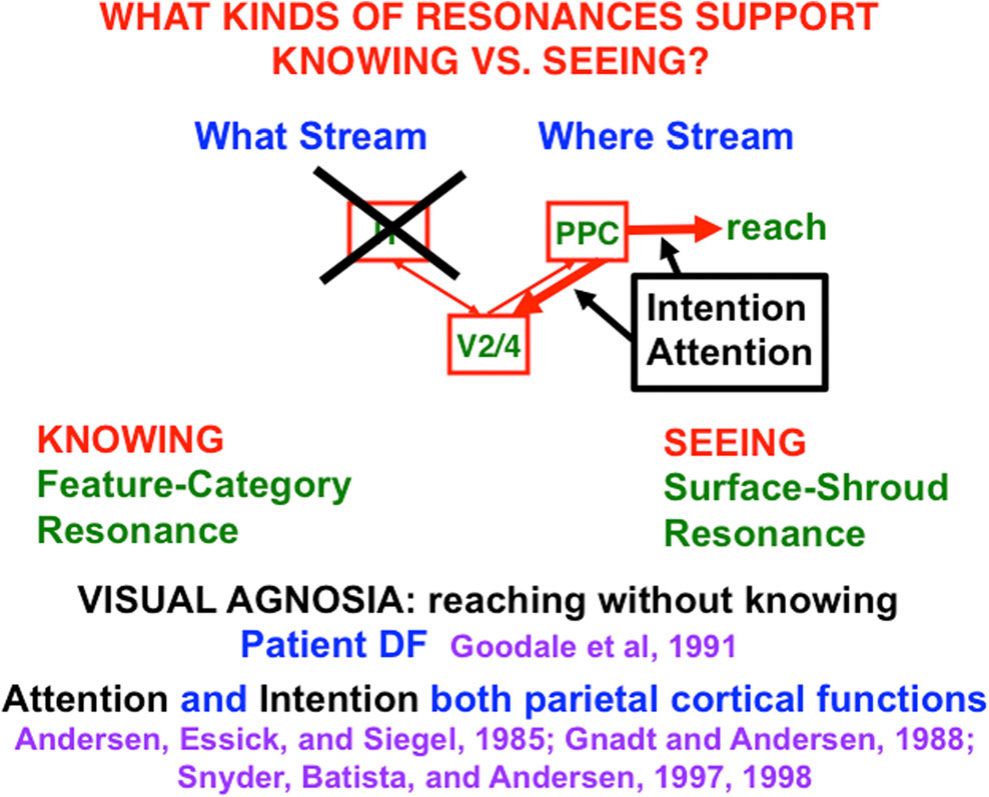
When a feature–category “knowing” resonance is lesioned, the corresponding surface–shroud “seeing” resonance can still trigger an action, as occurs during visual form agnosia. This figure also diagrams the distinction between the roles of PPC in controlling top-down spatial attention versus the intention to move

Figure 24 sheds light on what can go wrong during visual form agnosia. If an IT lesion prevents a feature–category resonance from forming, the surface–shroud resonance that is activated by a given object can still be intact. As noted in Section 2, the activated cells in the PPC can then also be used to direct an accurate eye movement or reach. Patients who exhibit visual agnosia often cannot recognize basic properties of object shape. They can, as in the case of patient D. F., nonetheless carry out accurate reaches to these objects (Goodale & Milner, 1992; Goodale et al., 1991). Many other clinical data have been explained by such resonances. See Franklin and Grossberg (2017), Grossberg (2017a, 2017b), and Grossberg and Kishnan (2018) for discussions of some of them.

**Fig. 25:**
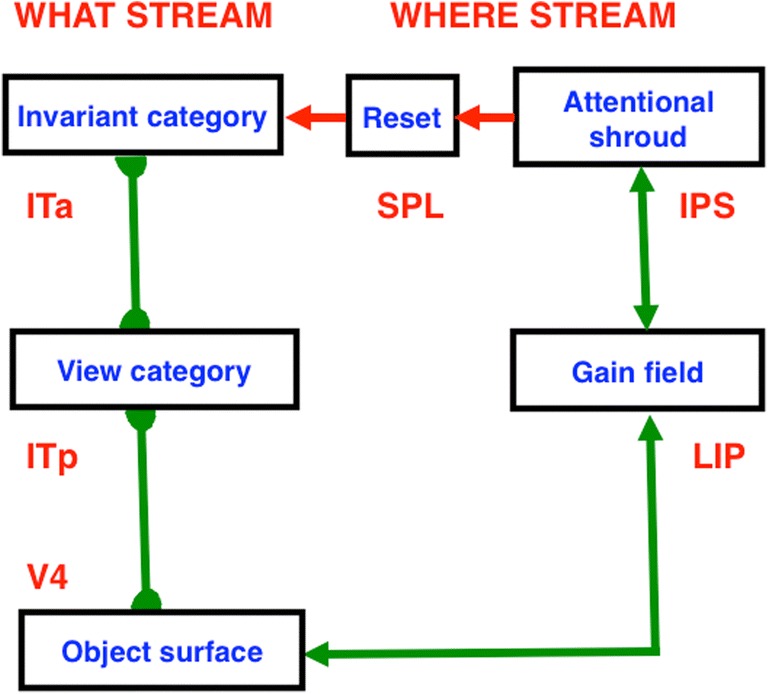
Learning of view-invariant categories in the “what” cortical stream is regulated by surface–shroud resonances in the “where” cortical stream. The surface–shroud resonance between V4 and IPS maintains sustained spatial attention upon an object surface at the same time that it prevents an emerging invariant category in ITa from being reset as multiple view-specific categories in ITp are learned and associated with it as the eyes scan the attended object surface. See the text for details

Figure 24 also clarifies two distinct, but interacting, roles of the PPC: It can control spatial *attention* via top-down pathways to V4 and other visual cortical areas, at the same time that it regulates the *intention* to move, whether by looking at an attended object through regions like the lateral intraparietal area (LIP) or reaching toward it via a parietal reach region (PRR) that is medial and posterior to LIP (Andersen et al., 1985; Andersen, Snyder, Batista, Buneo, & Cohen, 1998; Gnadt & Andersen, 1988; Snyder et al., 1997, 1998).The remainder of this article proposes how the intentional role of PPC is translated into actions, starting with sequences of saccadic eye movements that focus upon the salient features of an attended object surface, and thereby drive learning of an invariant object category that can later support conscious recognition of all of these views. As will be discussed below, and illustrated by Fig. 25, this translation requires multiple levels of coordinated feedback processing between several brain regions.

## 11. Solving the view-to-object binding problem during free scanning of a scene

An invariant recognition category is one for which the same small set of cells responds to different views, positions, and sizes of an object’s retinal images. Learning invariant categories enables our brains to avoid the combinatorial explosion of memories and search times that would be needed if a different exemplar of each of an object’s retinal image had to be learned and searched, and how they could all be associatively linked to generate recognition responses, such as the name of the object.

In order to explain how invariant object categories could be learned, it was necessary to first propose a solution of the *view-to-object binding problem.* This problem arises because, as our eyes scan a scene, two successive eye movements may focus on different parts of the same object or on different objects. How does the brain avoid learning to erroneously classify views of different objects together, and do so without an external teacher? For example, suppose that the eyes sequentially scan a face, bird, and cloud in a natural scene. Why does not the brain learn to associate them all with the same invariant object category?

Surface–shroud resonances were discovered as a key brain design for regulating what exemplars in a scene could be associated through learning with an emerging invariant object category. Only after this regulatory role for a surface–shroud resonance in invariant category learning was articulated did it gradually became clear this was the type of resonance that I had been seeking for many years in response to my predictions that “all conscious states are resonant states” (e.g., Grossberg, 1980) and that “all consciously visible qualia are surface percepts” (e.g., Grossberg, 1994). Putting these two assertions together led to the question: What kind of resonance supports conscious percepts of visible qualia? How do we consciously see?

As modeling invariant category learning progressed, it became clear that surface–shroud resonances had the requisite properties. That realization enabled a deeper understanding how feature–category resonances for recognition interact with surface–shroud resonances for seeing, and how surface–shroud resonances for seeing select surface representations that could be used to direct looking and reaching, as in Figs. 23 and 24.

**Fig. 26:**
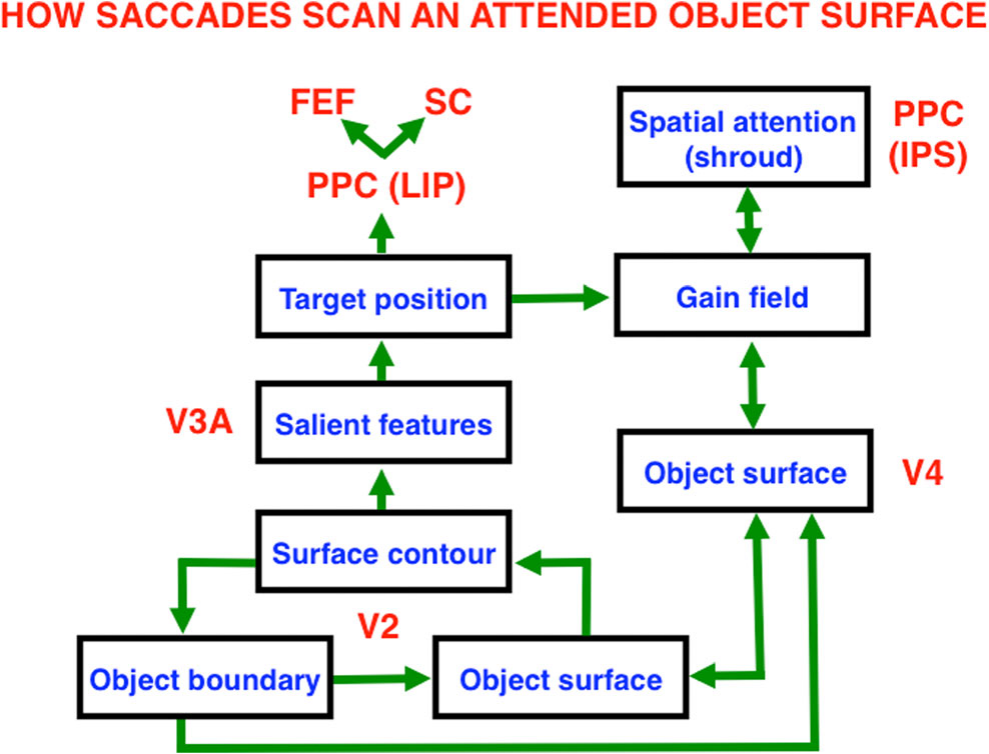
This circuit summarizes some of the key cortical processing stages that help to control sequences of saccadic eye movements that are directed to salient features of an attended object surface. See the text for details

This article will not fully describe how the 3-D ARTSCAN SEARCH family of models incrementally learns view-invariant, position-invariant, and size-invariant object recognition categories during free scanning of a scene with eye movements (Cao et al., 2011; Chang et al., 2014; Fazl et al., 2009; Foley, Grossberg, & Mingolla, 2012; Grossberg, Markowitz, & Cao, 2011; Grossberg, Srinivasan, & Yazdanbakhsh, 2014). The current article will focus on related and equally basic questions: Why do our eyes not saccade randomly around a scene? How do our eyes scan salient features on a single attended object surface for a while, even before we may have learned what the object is, so that multiple views of the object can, as a result, be bound together through learning into an invariant object category? Surface–shroud resonances provide answers to these questions by carrying out three coordinated functions that are summarized in Figs. 25 and 26:

First, a surface–shroud resonance maintains *sustained* spatial attention upon an object’s surface. Functional neuroimaging data in humans suggests that a region in the left posterior intraparietal sulcus (IPS) “may be involved in continuously maintaining the current state of attention” (Yantis et al., 2002), a conclusion that was also supported by Corbetta, Kincade, Ollinger, McAvoy, and Shulman (2000). Chiu and Yantis (2009) reported additional evidence for a surface–shroud resonance between V4 and PPC, notably “increased activation in extrastriate cortex and posterior intraparietal sulcus (IPS) contralateral to the locus of spatial attention” (p. 3933). Clinical patients cannot maintain sustained attention after they experience suitable parietal lesions, including lesions of the inferior parietal lobe, or IPL (Husain & Nachev, 2007; Rueckert & Grafman, 1998).

Second, an active shroud inhibits a population of tonically active reset cells in the parietal cortex (see Fig. 25). When the shroud shuts off, as occurs when spatial attention shifts from one object to another, these reset cells are disinhibited. They can then generate a transient burst of activation that inhibits any invariant object category in ITa that may be active at that time. Learning an invariant category of the newly attended object can then commence, without interference from the previously activate category.

This prediction was supported by experiments by Chiu and Yantis (2009) that used rapid event-related MRI in humans. These authors found that a shift of spatial attention evokes a transient domain-independent signal in the medial superior parietal lobule (SPL in Fig. 25) that corresponds to a shift in categorization rules. In the ARTSCAN model, collapse of an attentional shroud (spatial attention shift) in IPS disinhibits the parietal SPL reset mechanism (transient signal) that leads to inhibition in ITa of the active invariant object category and instatement of a new one (shift in categorization rules).

This transient parietal signal is “domain-independent” in the model because the parietal reset mechanism can be inhibited by spatial attention in PPC that focuses upon *any* object surface, and can reset *any* active invariant category in ITa when it is disinhibited. In other words, the category reset population of cells in medial SPL is predicted to receive converging inhibitory signals from many parts of PPC, and to emit diverging inhibitory signals to many parts of ITa. This experiment provides a useful marker for experimentally testing additional properties of the ARTSCAN model and its variants.

While maintaining spatial attention on an object surface, a surface–shroud resonance can also support saccadic eye movements that focus on salient features of the attended object surface. Figure 26 summarizes the circuits whereby these eye movements foveate different views of the object which, as summarized in Fig. 25, trigger learning of view-specific object categories in ITp, followed by associative learning with the emerging invariant object category in ITa. The next section explains how this is predicted to happen.

**Fig. 27:**
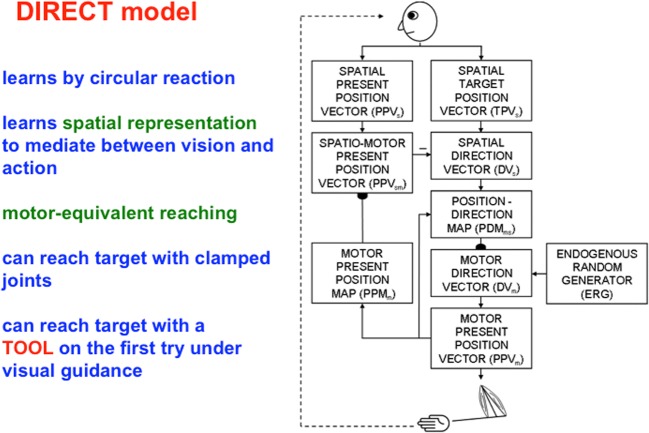
DIrection-to-Rotation Effector Control Transform, or DIRECT, model circuit mechanisms: An endogenous random generator, or ERG, energizes motor learning during a critical period of motor babbling. The ERG activates a motor direction vector (DVm) that moves the hand/arm via the motor present position vector (PPVm). As the hand/arm moves, the eyes reactively track the position of the moving hand, and compute a visually activated spatial target position vector (TPVs) and a spatial present position vector (PPVs). These vectors coincide during reactive tracking. Together they compute the spatial difference vector (DVs). This spatial computation, together with the mapping from spatial directions into motor directions, is the basis of motor-equivalent reaching properties. To compute them, the PPVs activates the spatiomotor present position vector (PPVsm), which is subtracted from the TPVs. Because the PPVs signal to the TPVs is slightly delayed, DVs can be computed. The PPVsm stage is one of two model stages where spatial (s) and motor (m) representations combine. A circular reaction (Piaget, 1945, 1951, 1952), is learned from spatial-to-motor and motor-to-spatial representations at the two adaptive pathways denoted by hemispherical synapses. The spatial direction vector (DVs) is hereby adaptively mapped into the motor direction vector (DVm) to transform visual direction into joint rotation. Adapted with permission from Bullock, Grossberg, and Guenther (1993)

## 12. Surface–shroud resonance enables saccades to foveate salient features of an attended object

### Surface contours compute salient features and attention pointers

In addition to achieving complementary consistency and initiating figure–ground separation, surface contours also compute target positions on an attended surface to which saccadic eye movements can be directed. This last property arises because a surface contour is generated by a *contrast-sensitive* on-center off-surround network that operates across space upon the filled-in surface contrasts of a given object, as was explained in Section 8.

As a result, the positions of salient features—such as positions where the curvature of the surface’s bounding contour changes quickly—are more active in a surface contour. The white circles at the corners of the filled-in rectangle at the bottom of Fig. 16 illustrate this property. Figure 20 also depicts how, using surface-to-boundary feedback signals, such a salience-sensitive surface contour strengthens its generative boundary while suppressing redundant boundaries at the same positions but at farther depths, thereby achieving complementary consistence and initiating figure–ground separation. These salient features have properties of what Cavanagh, Hunt, Afraz, and Rolfs (2010) called *attention pointers* because, as will now be explained using Fig. 26, these salient positions become the target positions of saccadic eye movements as the locus of attention shifts predictively across the object surface.

### From salient features to target positions: One role of V3A

Figure 26 summarizes the feedback loop that occurs within V2 between completed boundaries and filled-in surfaces, with surface contours closing the loop from surfaces in the thin stripes of V2 to boundaries in the interstripes of V2 (see Fig. 3). This is the feedback loop that is depicted in somewhat greater detail in Fig. 20. Figure 26 shows that outputs from V2 surface contours activate two parallel pathways. One pathway is the one depicted in Fig. 20. The other pathway chooses the most active position on a surface contour using a recurrent on-center off-surround winner-take-all network (Grossberg, 1973, 1980). The chosen position is the target position of the next saccade.

This transformation is predicted to occur between V2 and V3A. It must occur after V2 because it is in V2 that figure–ground separation occurs. The claim that V2 carries out figure–ground separation is supported by many experiments, notably the neurophysiological data about V2 in articles from the von der Heydt laboratory (e.g., O’Herron & von der Heydt, 2009; Qiu & von der Heydt, 2005; Qiu, Sugihara, & von der Heydt, 2007; von der Heydt, Zhou, & Friedman, 2000; Zhang & von der Heydt, 2010; Zhou, Friedman, & von der Heydt, 2000) that are given a unified explanation in Grossberg (2016a).

Regulating saccades to foveate on salient features of an attended object must occur after V2 because it is only after figure–ground separation occurs that attention *can* be focused on a prescribed object surface. V3A begins to transform visual representations to motor commands (Backus, Fleet, Parker, & Heeger, 2001; Caplovitz & Tse, 2007; Galletti & Battaglini, 1989; Nakamura & Colby, 2000). Indeed, Caplovitz and Tse (2007) have written that “neurons within V3A . . . process continuously moving contour curvature as a trackable feature . . . not to solve the ‘ventral problem’ of determining object shape, but in order to solve the ‘dorsal problem’ of what is going where” (p. 1179).

The target position commands from V3A also activate two parallel pathways (see Fig. 26). One pathway sends signals to the lateral intraparietal area (LIP) of the PPC, which, in turn, projects to the frontal eye fields (FEF) and the superior colliculus (SC) to generate saccadic eye movements to the chosen target position (Andersen, Brotchie, & Mazzoni, 1992; Bisley, Mirpour, Arcizet, & Ong, 2011; Blatt, Andersen, & Stoner, 1990; Goldberg, 2001; Nakamura & Colby, 2002; Olson & Colby, 2013; Paré & Wurtz, 2001; Snyder, 2000; Snyder et al., 2000). Along this route, LIP also projects to regions like the anterior intraparietal cortex, or AIP, that is used to control grasping movements (Battaglia-Mayer & Caminiti, 2009; Cohen & Anderson, 2002; Crawford, Medendorp, & Marotta, 2004; Nakamura et al., 2001), which will further discussed in Section 13.

### From salient features to gain fields and predictive remapping: Another role of V3A

The second pathway that receives target position commands is a *gain field* (Anderson et al., 1985, 1987; Andersen & Mountcastle, 1983; Deneve & Pouget, 2003; Fazl et al., 2009; Gancarz & Grossberg, 1999; Grossberg & Kuperstein, 1986; Pouget, Dayan, & Zemel, 2003) that operates between surface representations in V4 and spatial attentional shrouds in PPC. This gain field is a population of cells that is activated by target position signals and used to transform the retinotopic coordinates of an attended surface into the head-centered coordinates of its attentional shroud.

Why are shrouds computed in head-centered coordinates? The need for this arises from the fact that we consciously see visual surface qualia that are computed in *retinotopic coordinates*. In other words, we see whatever the eyes currently foveate in the center of our view, with previously foveated parts of a scene shifted to positions that lie in a direction opposite to that of the last eye movement. This state of affairs raises the following question: When a large eye movement occurs on an object surface, why does not the newly foveated position sometimes lie off positions of the shroud, thereby causing a collapse of the shroud as it does so? Such a collapse would disinhibit category reset cells, which can then inhibit the emerging invariant category, thereby preventing invariant object category learning from proceeding. Somehow the currently active shroud must remain stable as the eyes explore the surface of one object. The ARTSCAN model proposes that this is accomplished by computing shrouds in *head-centered coordinates* that do not move when the eyes move. The transformation from an attended surface in retinotopic coordinates to its attentional shroud in head-centered coordinates is accomplished by a gain field.

### Predictive remapping keeps the shroud in stable head-centered coordinates during saccades

The (target position)-to-(gain field) signals that update a head-centered shroud occur very quickly, before an eye movement is complete, to preserve the shroud’s head-centered representation during the eye movement. This process is called *predictive remapping*. Predictive remapping describes neurophysiological data about how parietal representations are updated by intended eye movements (Duhamel, Colby, & Goldberg, 1992; Gottlieb, Kusunoki, & Goldberg, 1998; Mathot & Theeuwes, 2010; Melcher, 2007, 2008, 2009; Saygin & Sereno, 2008; Sommer & Wurtz, 2006; Tolias et al., 2001; Umeno & Goldberg, 1997).

### Both retinotopic and spatial coordinates are needed during active vision

How the visual world appears to remain stable as our eyes actively scan a scene requires interacting combinations of retinotopic and head-centered, or spatial, representations. Functional neuroimaging (fMRI) data of Burr and Morrone (2011) illustrate this subtlety: “We firstly report recent evidence from imaging studies in humans showing that many brain regions are tuned in spatiotopic [head-centered] coordinates, but only for items that are actively attended” (p. 504). These data are consistent with properties of “attention pointers” that rapidly update gain fields via predictive remapping to maintain spatiotopic shroud stability during eye movements that scan an attended object, even while the conscious visual representation of the object surface is computed in retinotopic coordinates that move around with the eyes.

### Exploring an attended object surface with saccades: Why eyes do not move randomly

Putting together all of these observations provides an explanation of how our eyes can explore salient features of an attended object using sequences of saccadic eye movements. Figure 26 helps to keep the relevant interactions in mind. First suppose that a surface–shroud resonance is active between V4 and the IPS in the PPC. Due to the top-down excitatory signals to the attended surface representation in V4, the contrast of this surface is increased (see Fig. 22). As a result, its surface contours are also strengthened due to the contrast-sensitivity of the on-center off-surround network that generates them. The salient feature positions on these surface contours are correspondingly strengthened (see Fig. 16), thereby enabling these positions to more easily win the competition to determine the next target position of a saccade (see Figs. 26).

After a target position is chosen, it generates a saccade command to LIP and subsequent saccadic movement centers such as the frontal eye fields (FEF) and the superior colliculus (SC), while also rapidly updating the gain field that will keep the attentional shroud stably maintained in head-center coordinates when this eye movement is executed (see Fig. 26). As the target position generates these excitatory output signals, it also sends an inhibitory signal back to its source to prevent its perseverative performance. This kind of self-inhibition, or inhibition of return, has often been used in neural models of how saccade sequences are recalled (e.g., Grossberg & Kuperstein, 1986, Chapter 9; Silver, Grossberg, Bullock, Histed, & Miller, 2011), as well as, more generally, in models of how sequences of stored items in cognitive, motor, and spatial working memories are recalled (e.g., Grossberg & Pearson, 2008). Then, the next most active surface contour position can be chosen, and the saccadic cycle repeats itself until all the attended salient features are foveated, or the surface–shroud collapses.

Surface–shroud collapse can occur because the transmitters that multiplicatively gate the bottom-up and top-down excitatory signals that maintain the resonance habituate in an activity-dependent way, and/or the last saccade brings the eye closer to another object that can generate a stronger, nonhabituated, surface–shroud resonance. In this way, the eyes can continue to search different objects in a scene, and can inspect salient cues on each item before saccading to the next one. This search cycle coordinates processes of spatial and object attention, figure–ground separation, predictive remapping, invariant object category learning, and visual search.

This cycle also shows why saccades do not just randomly explore a novel scene. If they did, it would not be possible to learn view-invariant object categories. The ability of saccades to sequentially explore different views of an attended object even in novel scenes has been supported by Theeuwes, Mathot, and Kingstone (2010), whose psychophysical data show that “the eyes prefer to stay within the same object” (p. 597).

### Both transient and sustained parietal representations regulate attention

Different parts of PPC operate with different time scales that vary between sustained and transient. Sustained attention occurs between the inferior parietal sulcus (IPS) and V4 during a surface–shroud resonance (Chiu & Yantis, 2009; Corbetta et al., 2000: Yantis et al., 2002). The lateral intraparietal area (LIP) begins the conversion of a target position command into a saccade, and is reset to instate the next command even while the surface–shroud resonance persists. Finally, when the surface–shroud resonance does collapse, this shift of spatial attention causes a transient parietal reset burst in the medial superior parietal lobule (SPL; Chiu & Yantis, 2009).

## 13. From head-centered looking to body-centered motor-equivalent reaching sequences and tools

The circuits in Figs. 20, 25, and 26 clarify how sequences of salient target positions in head-centered coordinates can be chosen as an individual pays spatial attention to, and inspects, a novel or familiar object, leading to invariant object category learning, recognition, and visual search. Once these basic insights are available, they can be combined with other, compatible, modeling studies that explain, in addition, how head-centered spatial coordinates are transformed through learning into body-centered spatial coordinates both to control movement-invariant shrouds for invariant object category learning, as well as to control arm reaching movements to the same attended positions in space to which the eyes move (Y. E. Cohen & Andersen, 2002; Deubel, Schneider, & Paprotta, 1998; Schiegg, Deubel, & Schneider, 2003; Schneider & Deubel, 2002). How such a body-centered representation may be learned in real time using outflow neck position signals in addition to the outflow eye target position signals in Fig. 27 has been modeled in Guenther, Bullock, Greve, and Grossberg (1994). These body-centered spatial representations may be used to learn to control *motor-equivalent* arm movements. The DIRECT model of Bullock, Grossberg, and Guenther (1993) models motor-equivalent reaches that are accurate on the first try, even if the elbow is clamped at a fixed angle, just so long as the target is still within the arm’s workspace. They are also accurate on the first try if the target is reached with a tool under visual guidance, without measuring the length of the tool or its orientation in the hand, despite the fact that the tool constitutes an additional “limb” that has been added to the hand without any additional learning. The DIRECT model circuit is shown in Fig. 27.

Such a spatial affordance for tool use arises automatically in the model after it learns a representation of the space around it using a *circular reaction*, which is a principal way that reaching behaviors are learned in children (Piaget, 1945, 1951, 1952). It is called a “spatial” affordance for tool use because a representation of the space around the child is learned, and this spatial representation is downloaded into a command to move any limb to the desired target position. The human ability to use tools may thus have arisen from basic properties of how visually guided reaches in space are learned.

All babies normally go through a *babbling phase*, and it is during such a babbling phase that a circular reaction can be learned. During a visual circular reaction, babies endogenously babble, or spontaneously generate, hand/arm movements to multiple positions around their bodies. As their hands move in front of them, their eyes automatically, or reactively, look at their moving hands. While the baby’s eyes are looking at its moving hands, the baby learns an associative map from its hand positions to the corresponding eye positions, *and* from eye positions to hand positions. Learning of the map between eye and hand in both directions constitutes the “circular” reaction.

After map learning occurs, when a baby, child, or adult looks at a target position with its eyes, this eye position can use the learned associative map to activate a movement command to reach the corresponding position in space. If the volitional will to act is activated by opening the correct basal ganglia gate, then the selected hand/arm can reach to the foveated position in space under volitional control.

The DIRECT model begins to learn a circular reaction that is energized by an Endogenous Random Generator, or ERG (see Fig. 27). During the circular reaction, DIRECT learns how to combine the target position on the retina, the position of the eyes in the head, and the position of the head in the body into a representation of the position of the target in *space*. This spatial position can then be used to learn how to accurately reach with any of several motor effectors, which is the property of motor-equivalence, as well as with a tool. DIRECT hereby demonstrates how the *spatial affordance* for tool use, one of the most important foundations of human societies, is an automatic consequence of a brain’s ability to learn a circular reaction for motor-equivalent reaching in space. The caption of Fig. 27 explains the model properties that accomplish this.

This foundation enables the learning of sensory-motor skills. *Sequences* of eye saccades or arm reaching movements may be temporarily stored in an item-order-rank working memory in the prefrontal cortex before they are unitized through learning as sequence categories, plans, or *list chunks* by a masking field network (M. A. Cohen & Grossberg, 1986, 1987; Grossberg & Pearson, 2008; Silver et al., 2011). Such an item-order-rank working memory can store sequences of items or events that are repeated, as in the list ABACBD. Feedback interactions between an item-order-rank working memory and a masking field list chunking network enable stable learning of list chunks that can selectively respond to stored sequences of variable length. Activation of such a list chunk can read out previously learned sequences of skilled arm movements into working memory, from which they can be rehearsed under volitional control at variable speeds.

Cognitive–emotional interactions that are sculpted during reinforcement learning and incentive motivational learning enable the choice of that list chunk which, in the current context, controls the arm movement sequence that is most likely to acquire a valued goal in the current environment (e.g., Dranias, Grossberg, & Bullock, 2008).

Huang and Grossberg (2010) have, moreover, shown how the spatial positions and objects that have previously been searched in a scene can be stored in parallel spatial and object working memories that enable subsequent movement choices to use the context of previous sequences of choices to move to the best positions and objects in a familiar scene. This ARTSCENE Search model shows how spatial working memories in parahippocampal cortex and dorsolateral prefrontal cortex interact with object working memories in perirhinal cortex and ventrolateral prefrontal cortex to realize these properties. Such concepts have enabled the ARTSCENE Search model to quantitatively simulate all the major types of data from the psychophysical literature on contextual cueing.

The most advanced model of how action sequences may be controlled by cognitive and cognitive–emotional processes is the Adaptive Resonance Theory, or pART, model (Grossberg, 2018), which offers a unified neural theory of the prefrontal cortex and its functions. The pART combines all of the above properties, in addition to explaining how working memory storage in prefrontal cortex becomes *selective* and only enables task-relevant events to influence cognitive processing and action choices. The scope of pART is illustrated by the following summary of its properties.

The pART model explains and simulates how prefrontal cortices play an essential role in working memory and cognitive–emotional processes through interactions with multiple brain regions. Prefrontal properties of recent neurobiological data about desirability, availability, credit assignment, category learning, and feature-based attention are explained. These properties arise through interactions of orbitofrontal, ventrolateral prefrontal, and dorsolateral prefrontal cortices with the inferotemporal cortex, perirhinal cortex, parahippocampal cortex, ventral bank of the principal sulcus, ventral prearcuate gyrus, frontal eye fields, hippocampus, amygdala, basal ganglia, hypothalamus, and visual cortical areas V1, V2, V3A, V4, middle temporal cortex, medial superior temporal area, lateral intraparietal cortex, and posterior parietal cortex.

Model explanations also include how the value of visual objects and events is computed, which objects and events cause desired consequences and which may be ignored as predictively irrelevant, and how to plan and act to realize these consequences, including how to selectively filter expected versus unexpected events, leading to actions toward, and conscious perception of, expected events. Modeled processes include reinforcement learning and incentive motivational learning, object and spatial working memory dynamics, and category learning, including the learning of object categories, value categories, object-value categories, and sequence categories, or list chunks.

### Multiple prediction error processes in the brain and in technology

The pART model includes a significant role for the basal ganglia in regulating brain dynamics, including how the substantia nigra pars compacta (SNc) and related areas can regulate learning in response to unexpected outcomes, or prediction errors; and how the substantia nigra pars reticulata (SNr) and related areas can regulate the opening and closing of gates, by activating and deactivating volitional GO signals, that regulate what thoughts, feelings, and actions will actually be realized. In so doing, pART builds upon a sequence of previous detailed modeling studies of these basal ganglia functions, and the data that they have explained and predicted (e.g., Brown, Bullock, & Grossberg, 1999, 2004; Dranias et al., 2008; Grossberg, 2016b; Grossberg, Bullock, & Dranias, 2008; Grossberg & Kishnan, 2018).

These articles also clarify that multiple brain regions use predictive errors to guide new learning. In addition to the basal ganglia, such brain regions include the thalamocortical and corticocortical feedback circuits, interacting with brain regions like the nonspecific thalamus and hippocampus, that enable ART circuits to learn new recognition categories in response to novel or unexpected events (Carpenter & Grossberg, 1993; Grossberg & Versace, 2008). The kinds of prediction error that are computed in the basal ganglia, nonspecific thalamus, and hippocampus are different from the mismatches that can drive motor learning per se in the parietal and motor cortices (e.g., see Table 1b).

Other models have also proposed how prediction error modulates cortical coupling (e.g., den Ouden, Daunizeau, Roiser, Friston, & Stephan, 2010) and have used a Bayesian hierarchical learner to describe the model’s online inference process. Sleep/wake manipulations in Bayesian Helmholtz machines also use Bayesian methods (e.g., Dayan & Hinton, 1996). The main utility of these models is in adaptive prediction applications. In contrast, the biological neural models that are described herein enable a detailed understanding of the neural architectures that can rapidly learn and perform such inferences in changing environments that are filled with unexpected events, while also solving the stability–plasticity dilemma along the way, and providing unified explanations and predictions of large amounts of interdisciplinary data. Because they are fully specified mathematically, these models can also be used in applications, as have many others that my colleagues and I have developed (cf. http://techlab.bu.edu/resources/articles/C5).

## 14. Concluding remarks

This article summarizes some basic reasons why feedback processes operate at all levels of the cerebral cortex and thalamus. To illustrate the general prediction that “all conscious events are resonant events,” the article has described some of the main cortical processing stages that enable computationally complementary boundary and surface representations to be completed and filled-in via a process of hierarchical resolution of uncertainty. Then a surface–shroud resonance consciously “lights up” a surface representation that is complete, context-sensitive, and stable enough to be used to direct successful looking and reaching behaviors. This analysis also distinguishes between feature–category resonances for knowing, or recognition, and surface–shroud resonances for seeing, and suggests how we can know about familiar objects that we see due to synchronization of these resonances via shared circuits in prestriate cortical areas V2 and V4.

Either of these kinds of resonances can generate top-down expectation signals from V4 to earlier cortical stages, even to the lateral geniculate nucleus (Gove, Grossberg, & Mingolla, 1995; Murphy & Sillito, 1987; Sillito, Jones, Gerstein, & West, 1994). As noted in Section 1, due to the way in which object attention works—via top-down, modulatory on-center, off-surround networks that embody the ART Matching Rule (Bhatt, Carpenter, & Grossberg, 2007; Carpenter & Grossberg, 1987, 1991; Grossberg, 1980, 2013), which is also sometimes called “biased competition” (Desimone, 1998; Kastner & Ungerleider, 2001; Reynolds & Heeger, 2009)—these top-down signals can select cell activations that are consistent with the resonating surface representation, while suppressing cell activations that are not, thereby selecting those lower-level representations that are compatible with the chosen action.

The article also describes how feedback interactions among multiple cortical areas can direct sequences of saccadic eye movements to foveate salient features of an attended surface. Attention upon the surface is sustained via a surface–shroud resonance, which can also be consciously seen as a result. These cortical regions include both IPS and LIP within PPC, as well as V2, V3A, and V4 within the prestriate visual cortex. The different foveated object views can then trigger learning of view-specific object categories in cortical areas like ITp via feature–category resonances, which are then linked together by associative learning to create invariant object categories in cortical areas like ITa.

ART hereby provides a computational explanation of why both feature–category resonances and surface–shroud resonances are needed. In particular, perceptual and cognitive processes in the “what” ventral processing stream use excitatory matching and match-based learning (see Table 1b) to learn categorical representations of objects and events in the world using feature–category resonances. Match-based learning solves the stability–plasticity dilemma and can occur quickly without causing catastrophic forgetting, much as new faces can be learned quickly without forcing unselective forgetting of familiar faces.

Such match-based learning supports the creation of category representations at higher cortical levels that are increasingly invariant under changes in an object’s views, positions, and sizes. That is, match-based learning can support *invariant* category learning (see Section 6), which enables learning to categorize the world without causing a combinatorial explosion of memories. However, positionally invariant object category representations cannot, by themselves, be used to manipulate objects at particular positions in space.

That is why complementary spatial and motor processes in the “where/how” dorsal cortical processing stream are needed to focus spatial attention upon and manipulate objects in space. These processes often use VAM-like inhibitory matching and mismatch learning (Section 5, and Table 1b) to continually update spatial maps and sensory–motor gains whereby to control looking or reaching behaviors (see Fig. 24). These inhibitory circuits cannot support an adaptive resonance, and thus do not generate conscious states.

Either excitatory or inhibitory matching and learning process in Table 1b is insufficient on its own to learn about the world and to effectively act upon it, but together they can. Perceptual and cognitive processes use excitatory matching and match-based learning to create self-stabilizing representations of objects and events that embody increasing expertise about the world, and conscious awareness of it. Complementary spatial and motor processes use inhibitory matching and mismatch learning to continually update spatial maps and sensory-motor gains to compensate for bodily changes throughout life. Together they provide a self-stabilizing perceptual and cognitive front end for conscious awareness and knowledge acquisition, which can intelligently manipulate more labile spatial and motor processes that enable our changing bodies to act effectively upon a changing world.
